# Combining Anisotropic ^119^Sn NMR and *Ab Initio* Calculations to Probe the Stereoactive Lone Pair in Sn(II) Metal–Organic Frameworks

**DOI:** 10.1021/acs.chemmater.6c00369

**Published:** 2026-06-15

**Authors:** Helena R. Loan, Ryan J. Bragg, Caitlyn F. Walton, Richard I. Walton, Michael A. Hope

**Affiliations:** Department of Chemistry, 2707 University of Warwick, Coventry CV4 7AL, U.K.

## Abstract

The coordination environments of several Sn­(II) metal–organic frameworks (MOFs) have been investigated using ^119^Sn solid-state NMR in combination with density functional theory (DFT) calculations. The Sn­(II) nucleus exhibits high chemical shift anisotropy (CSA) due to its lone pair, with spans of up to Ω = 1280 ppm. The CSA is very sensitive to the local coordination environment; however, large CSAs are challenging to measure, which is overcome using a combination of variable magic-angle spinning and static ultra-wideline BRAIN-CP-WCPMG experiments. In some cases, the spectra are further complicated by the presence of Sn–Sn *J* coupling. We measured the CSA of six Sn­(II) carboxylate MOFs, with between 1 and 3 crystallographically distinct Sn sites per structure. Comparing the experimentally measured ^119^Sn NMR tensors with those calculated from GIPAW DFT, we find good agreement with the isotropic shift, but DFT systematically underestimates the CSA. By accounting for the anisotropic effect of the Sn­(II) lone pair on the shielding tensor, the experimental CSAs can be accurately calculated. The combination of anisotropic NMR and DFT sheds light on the stereoactive Sn lone pair, which directs the material properties.

## Graphical abstract

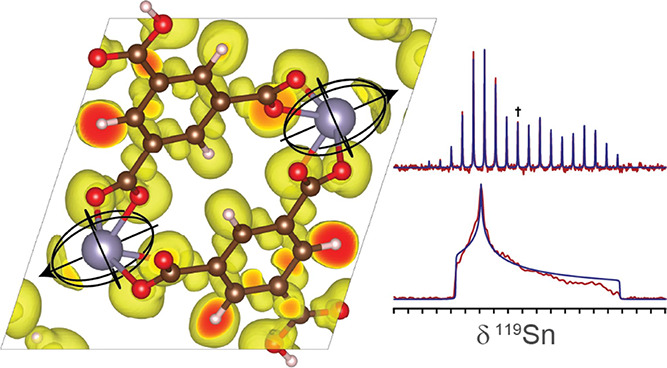

## Introduction

Metal–organic frameworks (MOFs) are promising materials for wide-ranging applications, such as gas separation, pollutant capture, energy storage, or catalysis.^1–7^ MOFs consist of metal cation nodes connected via organic linker molecules to form 2D or 3D structures that have the potential for porosity. The metal and organic molecules can be varied, allowing the materials to be optimized toward a desired function. Although Sn­(II) MOFs have been less studied than materials constructed from other metal cations, such a transition metals, they notably exhibit unusual structures due to the stereoactive Sn­(II) lone pair.^8^ Sn­(II) MOFs constructed from carboxylate ligands have been found to be remarkably stable toward moisture, they have even been crystallized under hydrothermal conditions.^9^ This makes these materials worthy of further study for multiple practical applications. A variety of Sn­(II) MOFs, including Sn_2_(DOBDC) (see below), have been reported as lithium-ion battery anodes^10–13^ and Sn­[(PDC)­(H_2_O)] has promising second harmonic generation properties.^14^ More generally, stereoactive lone pairs determine the photocatalytic, optoelectronic, and nonlinear properties of many functional materials.^15–17^

In the applications of MOFs, chemical species are often intercalated within the material, leading to local structural changes. To follow these changes and understand the functional mechanism requires a local structural probe, such as nuclear magnetic resonance (NMR) spectroscopy.^18–21^ Both the isotropic chemical shift, δ_iso_, and the chemical shift anisotropy (CSA) are sensitive to the coordination environment, i.e., coordination number, geometry, distance, and identity of nearest and next-nearest neighbors. The isotropic chemical shift is the orientationally averaged chemical shift (as observed in solution state NMR), while the chemical shift anisotropy describes the difference in chemical shift in different directions.

Sn­(II) coordination can be highly anisotropic due to the stereoactive lone pair, which results in large CSAs for ^119^Sn. The CSA is therefore particularly useful for exploring the coordination environment, and changes on interaction with guest species. The magnitude of the CSA can be described by the span, Ω, with the deviation from axial symmetry described by the skew, κ (see Experimental Section for definitions).^22^ Sn has three NMR-active isotopes, but ^119^Sn NMR spectroscopy is the most common. ^119^Sn NMR has been applied to a wide range of materials that contain both Sn­(II) and Sn­(IV) oxidation states,^23–29^ although experimental difficulties can arise due to the air sensitivity of many Sn­(II) compounds, as well as the challenge of measuring large CSAs.

To interpret experimental NMR parameters and directly relate them to structure, *ab initio* chemical shift calculations are commonly used, typically based on density functional theory (DFT); this combination is known as NMR crystallography.^30–33^ However, meaningful comparison between calculated and experimental CSAs is more challenging than for isotropic shifts, reflecting both the sensitivity of CSA to structure and dynamics that may not be captured by the calculations, and the greater uncertainty associated with the experimental determination of CSA tensor parameters.^34^ Furthermore, there are challenges in applying DFT methods to heavy elements arising from relativistic effects, and there are few examples of CSA calculations of heavier elements.^35,36^

Here we report the acquisition of the ^119^Sn NMR spectra of six different Sn­(II) carboxylate MOFs. To measure the large CSAs we use both variable-rate magic-angle spinning (MAS) and static ultra-wideline methods (namely BRAIN-WURST-CPMG, see below). We then calculate the ^119^Sn chemical shift tensors, benchmarking different exchange-correlation functionals, and by careful treatment of the whole tensor, we can accurately reproduce both the isotropic chemical shift and the CSA.

## Results and Discussion

Six recently reported Sn (II) MOFs based on different benzene carboxylate linkers (Figure 1) were hydrothermally synthesized,^8,37,38^ and their purity confirmed by powder X-ray diffraction (PXRD) compared to the simulated patterns from the reported crystal structures, as shown in Figure S1. These materials all have relatively dense frameworks, with their structures and connectivity described in each section below.

**Figure 1:**
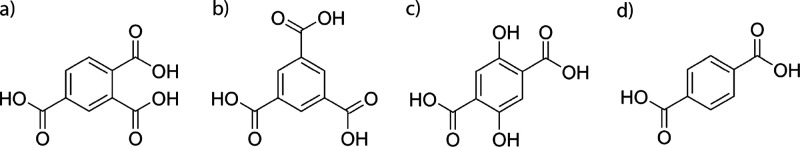
Skeleton structures of the acid form of the linkers used as reagents: (a) benzene-1,2,4-tricarboxylic acid (H_3_-1,2,4-BTC), (b) benzene-1,3,5-tricarboxylic acid (H_3_-1,3,5-BTC), (c) 2,5-dihydroxy-benzene-1,4-dicarboxylic acid (H_4_-DOBDC), and (d) benzene-1,4-dicarboxylic acid (H_2_-1,4-BDC).

### Sn­(H-1,2,4-BTC)

Sn­(H-1,2,4-BTC) has one unique Sn­(II) environment (Figure 2a, inset) with five oxygens coordinated in a distorted pyramidal geometry. The Sn atoms are linked via Sn–O bonds, (defined here as ≤3 Å separation) to give a zigzag inorganic chain that runs parallel to the *b* axis. These chains are bridged by H-benzene-1,2,4-tricarboxylate (H-1,2,4-BTC, Figure 1a) in the *a* direction to form a 2D coordination polymer. The linker contains one protonated carboxylate group which forms a carboxylic acid dimer with a neighboring ligand to hold the MOF layers together (Figure 2a).

**Figure 2:**
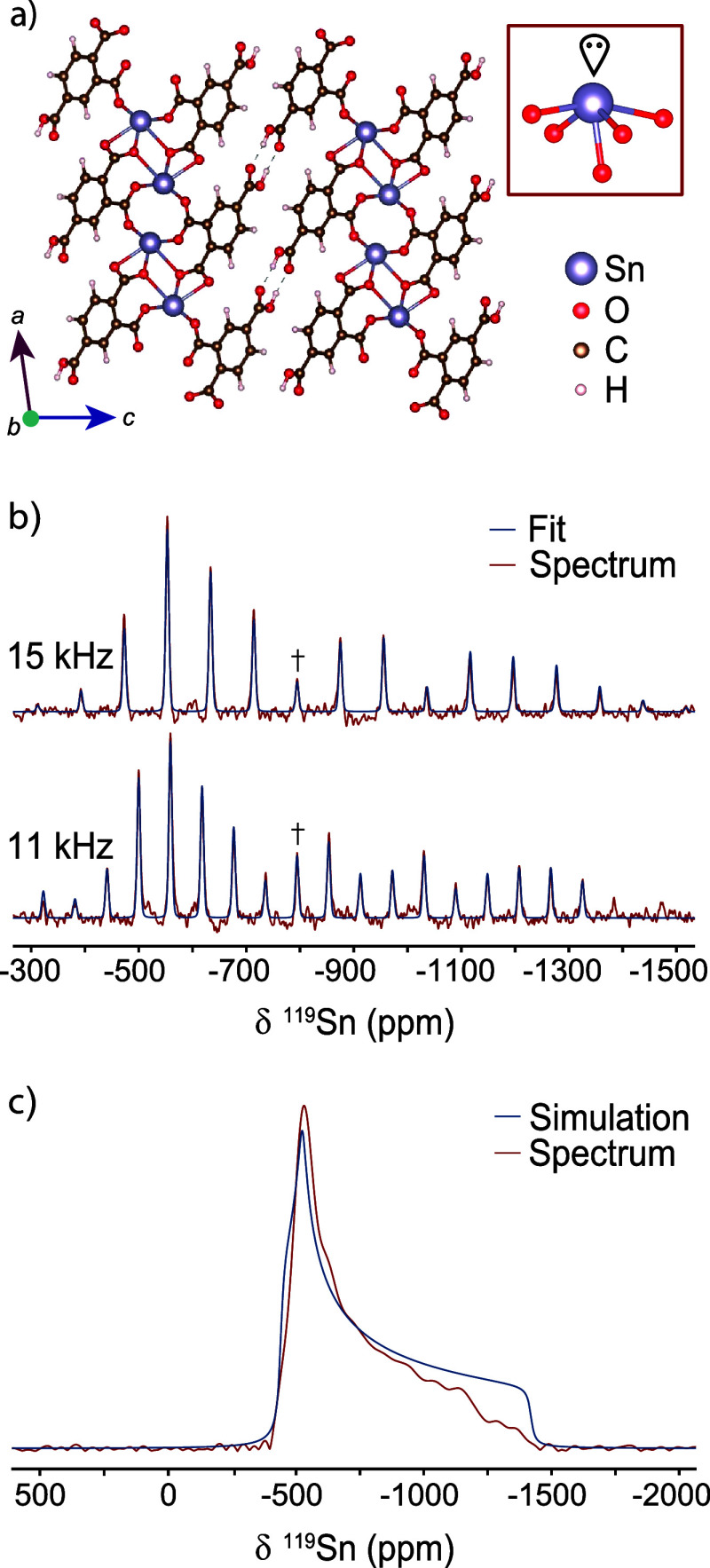
Structure and ^119^Sn NMR spectra of Sn­(H-1,2,4-BTC). (a) Structure in the *ac* plane showing the hydrogen bonding between −COOH groups of neighboring linkers. Inset of the unique Sn­(II) environment bonded to five oxygens in a distorted pyramidal coordination geometry.^8^ (b) ^119^Sn NMR spectra (11.7 T) at 11 and 15 kHz MAS using a one-pulse sequence, and fitted spectra with δ_iso_ = −795 ppm, Ω = 979 ppm, and κ = 0.82. The isotropic shift is indicated by †. (c) Static ^119^Sn NMR spectrum acquired using a BRAIN-CP-WCPMG pulse sequence and simulated spectrum with the parameters from (b).

^119^Sn spectra were obtained at MAS speeds of 11 kHz and 15 kHz (Figure 2b). Comparing these spectra allows the isotropic shift of δ_iso_ = −795 ppm, to be determined (marked with †). To determine the CSA, the spinning sideband patterns of the two spectra were simultaneously fitted using ssNake^39^ (Figure 2b and Table 1), yielding a span of Ω = 979 ppm and a skew of κ = 0.82 (see Experimental Section for definitions). The large CSA reflects the anisotropic Sn environment comprising the coordinating oxide ions and the lone pair (Figure 2a), while the skew of close to 1 shows that the coordination geometry is close to axial.

**Table 1:** Fitted ^119^Sn CSA Parameters for the Six Sn­(II) MOFs Studied Here

MOF	CCDC number	δ_iso_ (ppm)	Ω (ppm)	κ
Sn(H-1,2,4-BTC)	2309735	–795	979	0.82
Sn(H-1,3,5-BTC)	2309734	–765	1205	0.70
Sn_2_(DOBDC)	2309736	–717	1068	0.76
Li_2_Sn_2_(1,4-BDC)_3_(H_2_O)_2_	785324	–555	998	0.96
Sn_2_(1,3,5-BTC)(OH)	2309733	–678	1154	0.84
		–773	1106	0.75
Sn_3_O(1,4-BDC)_2_	791590	–555	1241	0.98
		–581	1115	0.90
		–603	1224	0.70

Fitting MAS spectra gives a more accurate measurement of CSA than static spectra, when at least 5 sidebands are visible.^40^ However, these MAS direct excitation experiments suffer from low sensitivity due to the long ^119^Sn *T*_1_ relaxation time of 56 s (Table S1) and its relatively low natural abundance (8.59%);^23^ these spectra took 7.5 h to acquire with a signal-to-noise ratio (SNR) of ∼40. To improve sensitivity, a static ^119^Sn spectrum was acquired using a BRAIN-CP-WCPMG pulse sequence (see Experimental Section).^41–43^ The broadband adiabatic WURST pulse in BRAIN-CP enables cross-polarization (CP) to the broad ^119^Sn signal, which could not be effectively spin locked with a hard pulse; however, the frequency evolution under MAS interferes with the WURST spin lock, so that these experiments are most easily performed under static conditions. BRAIN-CP benefits from the greater magnetization of ^1^H and shorter ^1^H*T*_1_ (3.9 s, Table S1), yielding 20 times higher sensitivity (SNR per √time) and hence shorter acquisition times (13 min). The simulated static spectrum using the fitted CSA parameters from the MAS experiments reproduce the observed static line shape (Figure 2c). This lends confidence to the CSA values determined by fitting the MAS spectra, especially the skew, as it has been reported that MAS experiments fail to accurately determine the asymmetry when close to axiality (i.e., |κ| > 0.6).^44^ There is some missing intensity at lower frequency, which is ascribed to the relative orientation of the ^1^H–^119^Sn dipolar tensor and the ^119^Sn CSA tensor, resulting in less efficient CP for crystallites on the tail of the CSA pattern (Figure S2). Recently, it has been shown how to acquire BRAIN-CP-WCPMG spectra under MAS conditions.^45^ This enables an MAS spectrum to be acquired with high sensitivity (Figure S3) although further optimization may be required to directly extract CSA parameters.

### Sn­(H-1,3,5-BTC)

Sn­(H-1,3,5-BTC) also contains one unique Sn­(II) site with a disphenoidal geometry coordinated with four oxygens (Figure 3a, inset). The linker, H-benzene-1,3,5-tricarboxylate (H-1,3,5-BTC, Figure 1b), has two deprotonated carboxylate groups which each bind to two different Sn atoms, forming 1D tubular chains parallel to the *a* axis (Figure S4). The remaining protonated carboxylate group forms a carboxylic dimer, which holds the chains together (Figure 3a).

**Figure 3:**
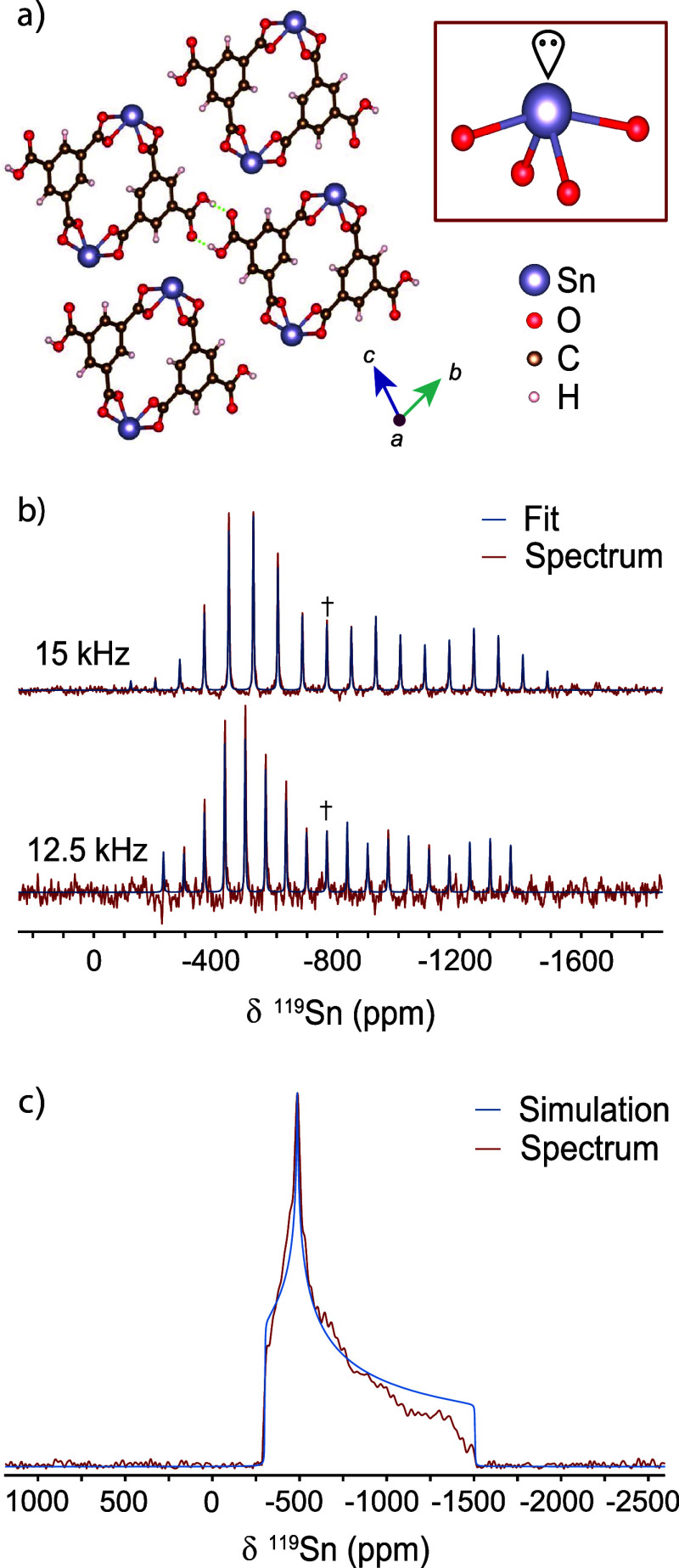
Structure and ^119^Sn NMR spectra of Sn­(H-1,3,5-BTC). (a) Structure in the *bc* plane showing the linker bonding to two pairs of Sn atoms and interchain carboxylic acid dimers. Inset of the unique Sn­(II) environment bonded to four oxygens in a disphenoidal geometry.^8^ (b) ^119^Sn NMR spectra (11.7 T) at 12.5 and 15 kHz MAS using a one-pulse sequence and fitted spectra with δ_iso_ = −765 ppm, Ω = 1205 ppm, and κ = 0.70. The isotropic shift is indicated by †. (c) Static ^119^Sn NMR spectrum using a BRAIN-CP-WCPMG pulse sequence and simulated spectrum with the parameters from (b).

^119^Sn spectra were obtained at MAS speeds of 12.5 and 15 kHz (Figure 3b) allowing the δ_iso_, −765 ppm, to be determined. Simultaneous fitting of the spinning sidebands yields a larger span of Ω = 1205 ppm and a skew of κ = 0.70 (Table 1). The static BRAIN-CP-WCPMG ^119^Sn spectrum provides a 14-fold higher sensitivity and agrees well with the simulated spectrum using the span and skew from the MAS experiments (Figure 3c).

### Sn_2_(DOBDC)

In Sn_2_(DOBDC), there is one unique four-coordinate Sn­(II) site with a distorted disphenoidal geometry, and the 2,5-dihydroxy-benzene-1,4-dicarboxylate acid (DOBDC^4–^) linker is fully deprotonated (Figure 1c). The Sn sites are bridged by pairs of phenoxide oxygens to create dimeric units with direct Sn–O–Sn bonds (Figure 4a). These units are connected in three dimensions by the DOBDC^4–^ linker in a herringbone arrangement (Figure S5).

**Figure 4:**
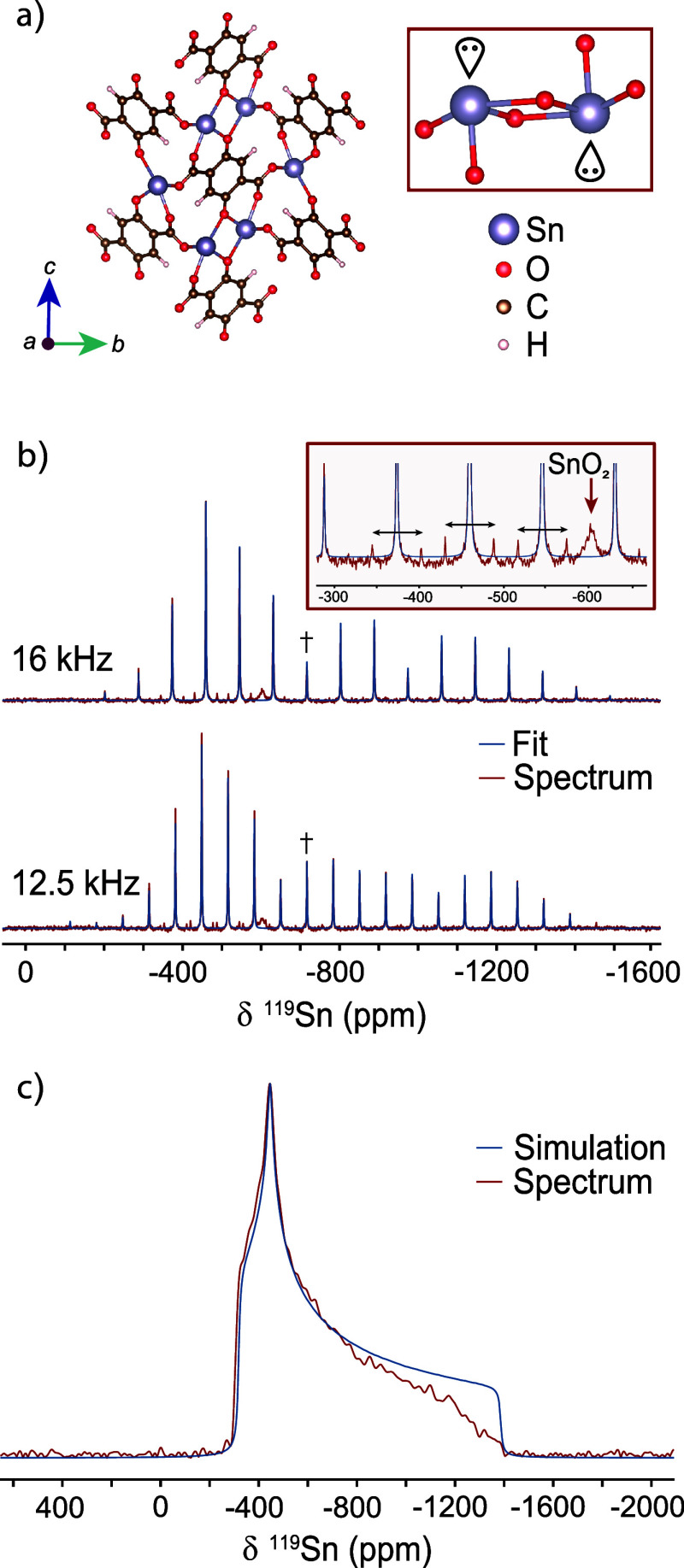
Structure and ^119^Sn NMR spectra of Sn_2_(DOBDC). (a) Dimeric units connected along the *a* axis to form a pseudolayered structure in the *bc* plane. Inset of the Sn dimer, with pairs of equivalent Sn sites bonded to four oxygens in a distorted disphenoidal geometry.^8^ (b) ^119^Sn NMR spectra (11.7 T) at 12.5 and 16 kHz MAS using a one-pulse sequence and fitted spectra with δ_iso_ = −717 ppm, Ω = 1068 ppm, and κ = 0.76. The isotropic shift is indicated by †. Inset showing the *J* = 10.7 kHz coupling between ^115/117^Sn–^119^Sn and SnO_2_ impurity at −603 ppm. (c) Static ^119^Sn spectrum using a BRAIN-CP-WCPMG pulse sequence and simulated spectrum with the parameters from (b).

Simultaneous fitting of the MAS spectra at 12.5 and 16 kHz (Figure 4b) gives δ_iso_ = −717 ppm, Ω = 1068 ppm, and κ = 0.76 (Table 1). As before a static spectrum was acquired which gave higher sensitivity and could be fit with the parameters from the MAS experiments (Figure 4b). On closer inspection of the ^119^Sn MAS spectra, satellite peaks can be seen (Figure 4b, inset) due to *J* coupling (10.7 kHz) of ^119^Sn and ^115/117^Sn pairs within the dimer. According to the binomial distribution, the probability of ^115/117^Sn being a nearest neighbor is 8%, which is approximately consistent with the observed satellite intensity of 7%. The *J* coupling of 10.7 kHz is larger than previously reported values of 2.6–8.2 kHz.^46,47^ Finally, there is evidence of a SnO_2_ impurity at −603 ppm, which was not visible in the PXRD (Figure S1d).

### Li_2_Sn_2_(1,4-BDC)_3_(H_2_O)_2_

In Li_2_Sn_2_(1,4-BDC)_3_(H_2_O)_2_ the unique Sn­(II) site is coordinated by a trigonal pyramid of oxide ions with three shorter Sn–O1 bonds (2.18 Å) as well as a trigonal arrangement of three longer Sn–O2 bonds (2.86 Å), such that it is coordinated by six oxygens in total (Figure 5a, inset). Each fully deprotonated 1,4-BDC ligand (Figure 1d) is bonded to two Sn atoms on either end of the ligand. Four interconnecting frameworks of [Sn_2_(1,4-BDC)_3_]^2–^ units come together to form a tetrahedron of Sn atoms, with the Sn lone pairs pointing toward the center (Figure 5a). The Li ions, located in the voids of the framework, bind the framework together through Li–O2 bonds to create a 3D structure (Figure S6).

**Figure 5:**
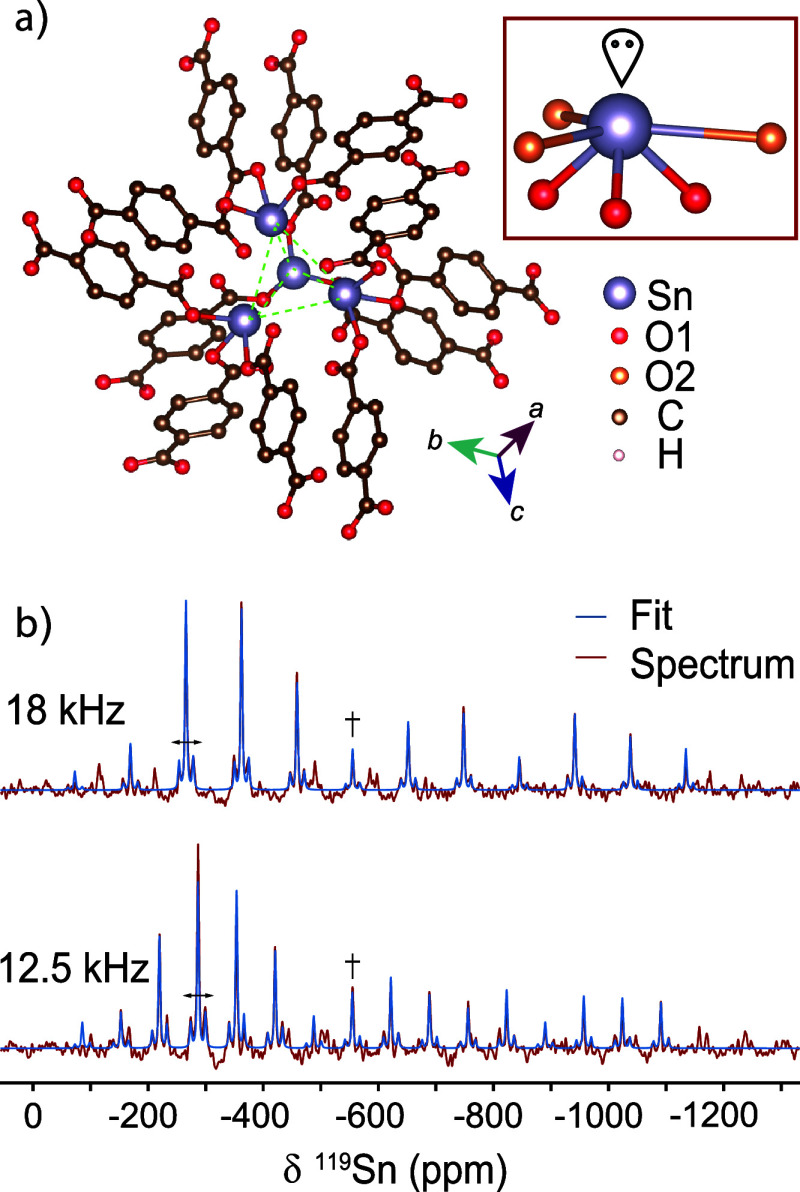
Structure and ^119^Sn NMR spectra of Li_2_Sn_2_(1,4-BDC)_3_(H_2_O)_2_. (a) Four (Sn_2_(1,4-BDC)_3_)^2–^ units form a [Sn_4_] tetrahedron. Inset of unique Sn­(II) environment bonded to six oxygens: three short bonds forming a trigonal pyramid and three longer bonds.^37^ (b) ^119^Sn NMR spectra (11.7 T) at 12.5 and 18 kHz MAS using a one-pulse sequence and fitted spectra with δ_iso_ = −555 ppm, Ω = 998 ppm, and κ = 0.96. Arrows correspond to *J* = 4.8 kHz coupling between ^115/117^Sn and ^119^Sn.

Simultaneous fitting of the ^119^Sn spectra at MAS rates of 12.5 and 18 kHz (Figure 5b) yields δ_iso_ = −555 ppm, Ω = 998 ppm, and κ = 0.96. A static ^119^Sn spectrum was unable to be acquired using a BRAIN-CP-WCPMG pulse sequence, which is tentatively ascribed to the presence of dynamic H_2_O within the sample causing the CP transfer to be ineffective. Satellite peaks are also apparent in this sample, corresponding to ^115/117^Sn–^119^Sn *J* coupling of 4.8 kHz. Surprisingly, coupling occurs through space via overlap of the Sn lone pairs which point toward each other in the tetrahedral unit, with a side length of 3.9 Å (Figure 5a). Each Sn has three nearest Sn neighbors, so the probability that one of those nearest neighbors is ^115/117^Sn is 20%, comparable to the experimental intensity of ∼23%. Sn–Sn *J* coupling due to overlap of lone pairs has also previously been observed between layers in SnHPO_4_ and SnO.^46,47^ Some minor impurity peaks are also visible in the MAS spectra which have not been assigned.

### Sn_2_(1,3,5-BTC)­(OH)

Unlike the materials so far discussed, Sn_2_(1,3,5-BTC)­(OH) has two unique Sn­(II) sites. Sn1 is coordinated to five oxygens in a disphenoidal geometry and Sn2 is coordinated to four oxygens with a trigonal pyramidal arrangement. These two Sn sites are connected via a bridging hydroxide and a deprotonated carboxylic acid group from the 1,3,5-BTC linker (Figure 6a, inset). The Sn2 sites are connected by hydroxide in infinite inorganic chains parallel to the *c* axis (Figure S7a). The linker then cross-links these chains to create a three–dimensional connected structure. This MOF has potential void space in the structure (Figure S7b); however, the apparent channels are not continuous to allow access to guest molecules, so it cannot be considered as a porous material.

**Figure 6:**
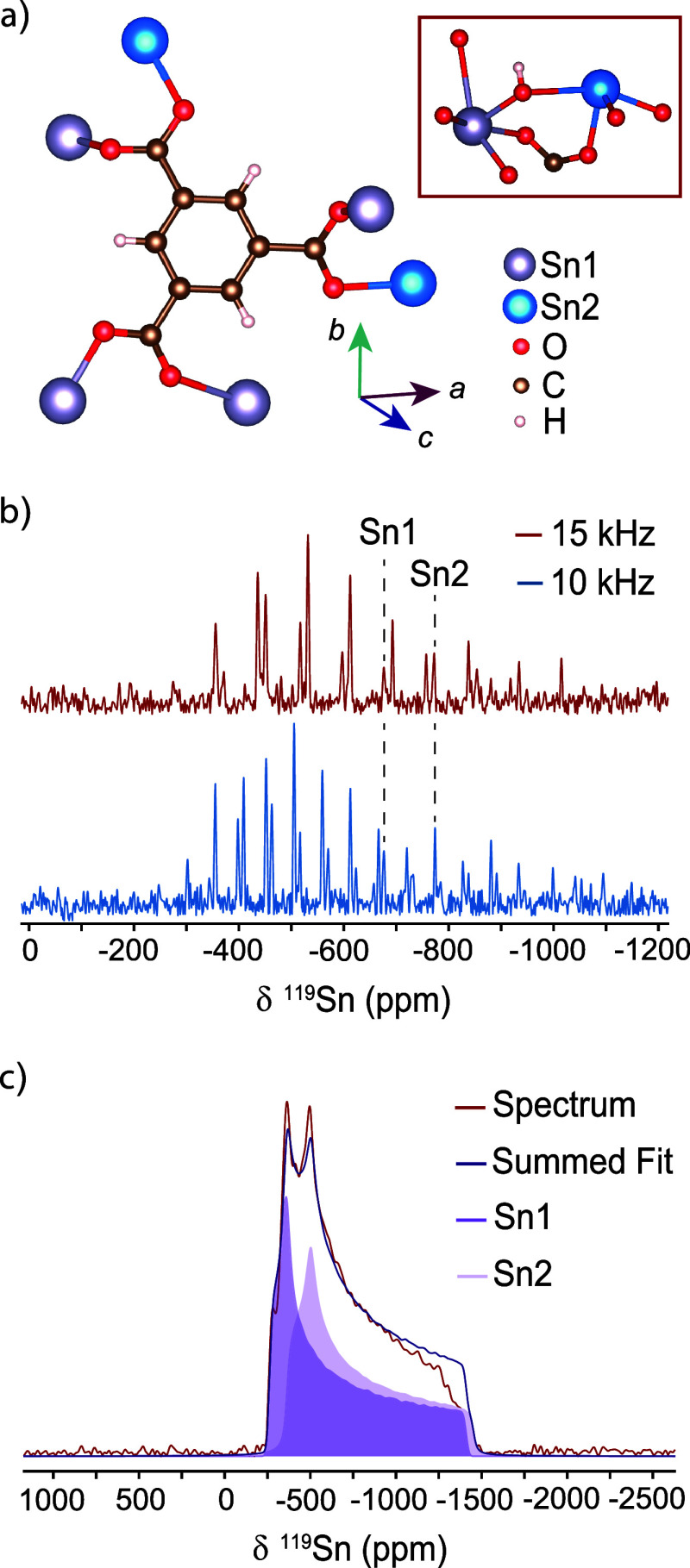
Structure and ^119^Sn NMR spectra of Sn_2_(1,3,5-BTC)­(OH). (a) Bonding of the linker to the Sn sites via the carboxylate. Inset of two different Sn­(II) environments, where Sn1 is bonded to five oxygens in a disphenoidal geometry and Sn2 is bonded to four oxygens with a trigonal pyramidal geometry. Both are connected via a hydroxide group.^4^ (b) ^119^Sn NMR spectra (11.7 T) at 10 and 15 kHz MAS using a WURST-CPMG pulse sequence. The peaks have been assigned using DFT calculations, see below. (c) Static ^119^Sn spectrum using a BRAIN-CP-WCPMG pulse sequence. Fit for this spectrum: δ_iso,1_ = −678 ppm, Ω_1_ = 1154 ppm, and κ_1_ = 0.84 and δ_iso,2_ = −773 ppm, Ω_2_ = 1106 ppm, and κ_2_ = 0.75.

The two distinct Sn sites make the ^119^Sn spectra more challenging to acquire. Single pulse experiments gave insufficient signal and so ^119^Sn MAS spectra were obtained using a WURST-CPMG pulse sequence at MAS speeds of 10 and 15 kHz (Figure 6b), allowing the two isotropic shifts of δ_iso_= −678 and −773 ppm to be determined. Spinning-sideband analysis could not be used on these spectra, since crystallites are not excited uniformly by the WURST pulses, changing the relative intensity of the sidebands. Instead, the static ^119^Sn spectrum acquired with BRAIN-CP-WCPMG was fitted (Figure 6c), fixing the isotropic shifts from the MAS spectra, to yield Ω = 1154 ppm and κ = 0.84 for the peak at −678 ppm, and Ω = 1106 ppm and κ = 0.75 for the peak at −773 ppm.

### Sn_3_O­(1,4-BDC)_2_

Sn_3_O­(1,4-BDC)_2_ has even greater complexity, with three distinct Sn sites. Sn1 is three-coordinate with a trigonal pyramidal geometry, while Sn2 and Sn3 are both four-coordinate with distorted disphenoidal geometries (Figure 7a, inset). The three Sn atoms are connected via an oxide to form [Sn_3_O] trimers that are linked via bridging carboxylate groups to form layers in the *ab* plane (Figure 7a and Figure S8). The 1,4-BDC ligand (Figure 1d) is completely deprotonated and cross-links adjacent layers of the trimers, to form a 3D framework structure (Figure 7a).

**Figure 7:**
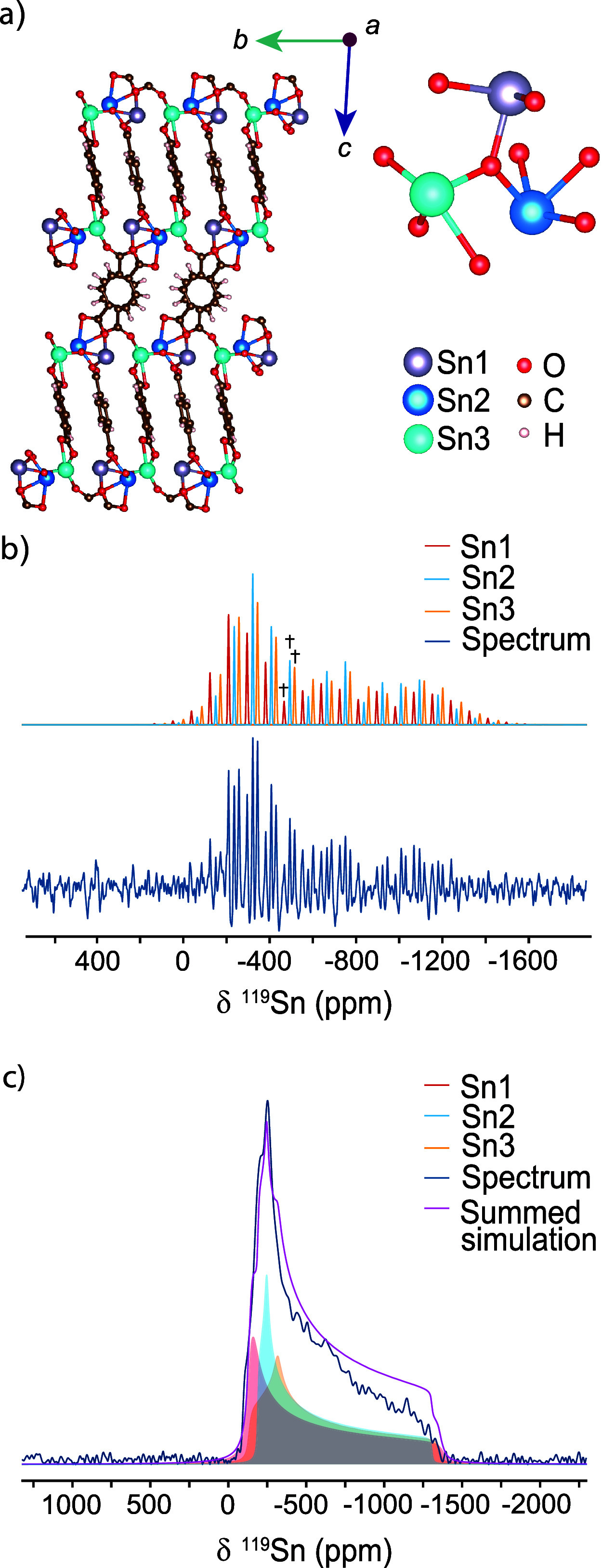
Structure and ^119^Sn NMR spectra of Sn_3_O­(1,4-BDC). (a) View along the *a* axis to show the layers in the *ab* plane are cross-linked via the ligand. Inset of the three different Sn­(II) environment: Sn1 is coordinated to three oxygens in a trigonal pyramidal geometry, and Sn2 and Sn3 are coordinated to four oxygens in a distorted disphenoidal geometry. All the Sn sites are connected by an oxide.^38^ (b) ^119^Sn NMR spectrum (11.7 T) at 16 kHz MAS using a one-pulse sequence and fitted spectrum with fit parameters in Table 2. The peaks have been assigned using DFT calculations, see below. (c) Static spectrum using a BRAIN-CP-WCPMG pulse sequence and simulated spectrum with the parameters from (b).

Simultaneous fitting of the one-pulse ^119^Sn spectra at MAS rates of 14, 15, and 16 kHz (Figure 7b and Figure S9) yields the shift parameters in Table 2. The low SNR of the MAS spectra means that there is greater uncertainty in the fitted values, however the static ^119^Sn spectrum acquired using BRAIN-CP-WCPMG (Figure 7c) corroborates the fits.

**Table 2:** Fitted ^119^Sn Parameters for Sn_3_O­(1,4-BDC) (Figure 7)

	δ_iso_ (ppm)	Ω (ppm)	κ
Sn1	–555	1241	0.98
Sn2	–581	1115	0.90
Sn3	–603	1224	0.70

### Comparison of ^119^Sn CSA Parameters

For all the Sn­(II) materials studied, the extracted CSA values are similarly large, with spans ranging from 979–1241 ppm. In contrast, literature span values for Sn­(IV) are usually <150 ppm.^48–51^ This suggests that the ^119^Sn CSA of Sn­(II) is predominantly influenced by the stereoactive lone pair. All skews are large and positive (κ > 0.7) because the chemical shift is smallest in the direction of the lone pair, and the two perpendicular directions are alike due to reasonably symmetric oxygen coordination. It is possible to draw qualitative trends between the skews of different Sn environments: disphenoidal geometries show the lowest κ (Sn­(H-1,3,5-BTC), Sn_2_(DOBDC), and Sn_3_O­(1,4-BDC)_3_ have skews between 0.70 and 0.76), and the samples where κ is very close to 1 have axially symmetric trigonal pyramidal geometries (Li_2_Sn_2_(1,4-BDC)_3_(H_2_O)_2_ and Sn_3_O­(1,4-BDC)_2_). To move beyond qualitative trends and relate the NMR parameters to the structure, DFT calculations are required.

### Calculating ^119^Sn NMR Parameters

To rationalize the observed ^119^Sn chemical shifts and CSAs, first-principles DFT calculations were performed starting from the previously reported experimental crystal structures.^8,37,38^ All calculations employed the CASTEP^32^ plane-wave code with the gauge-including projector augmented wave (GIPAW)^52^ algorithm (see Experimental Section). Two sets of calculations were performed: (1) nonrelativistic calculations using r^2^SCAN,^53^ a state-of-the-art nonempirical meta-GGA functional which has been shown to be substantially more accurate than GGA functionals for predicting lattice constants and ground-state structures of solids while maintaining numerical stability,^54,55^ and (2) relativistic calculations using the Perdew–Burke–Ernzerhof (PBE) GGA functional^56^ with the zeroth-order regular approximation (ZORA).^57,58^ This combined strategy enables an assessment of the relative importance of exchange–correlation sophistication and relativistic effects in calculating the NMR parameters for ^119^Sn as a moderately heavy element.^30,59–61^

Magnetic resonance computations yield the calculated absolute shielding tensor, σ^calc^, with principal components σ_11_^calc^, σ_22_^calc^, and σ_33_^calc^. The conversion between a principal component of the magnetic shielding tensor, σ_*ii*_^calc^, and the corresponding chemical shift component, δ_*ii*_^calc^, is given by

$$\delta_{ii}^{\mathrm{calc}}=\sigma_{ii}^{\mathrm{ref}}-\sigma_{ii}^{\mathrm{calc}}$$ 1

where σ_*ii*_^ref^ is the reference shielding, obtained by linear regression of the calculated shieldings against the experimental chemical shifts.^55^ Likewise, the isotropic shielding, σ_iso_^calc^ = (σ_11_^calc^ + σ_22_^calc^ + σ_33_^calc^)/3, is related to the isotropic shift by δ_iso_^calc^ = σ_iso_^ref^ – σ_iso_^calc^. Figure 8a compares the experimental isotropic ^119^Sn chemical shifts, δ_iso_^calc^, with the nonrelativistic isotropic ^119^Sn magnetic shieldings, σ_iso_^calc^, calculated using r^2^SCAN. There is a strong linear correlation with the gradient fixed to −1 (*R* ^2^ = 0.94, RMSD = 41.0 ppm), indicating that nonrelativistic DFT can accurately predict experimental ^119^Sn δ_iso_ values. Including scalar-relativistic effects via PBE+ZORA further improves the correlation (*R* ^2^ = 0.99, RMSD = 14.7 ppm; Figure 8b). These findings are consistent with prior reports showing that relativistic corrections improve the accuracy of calculated isotropic NMR parameters for ^119^Sn.^60,61^

**Figure 8:**
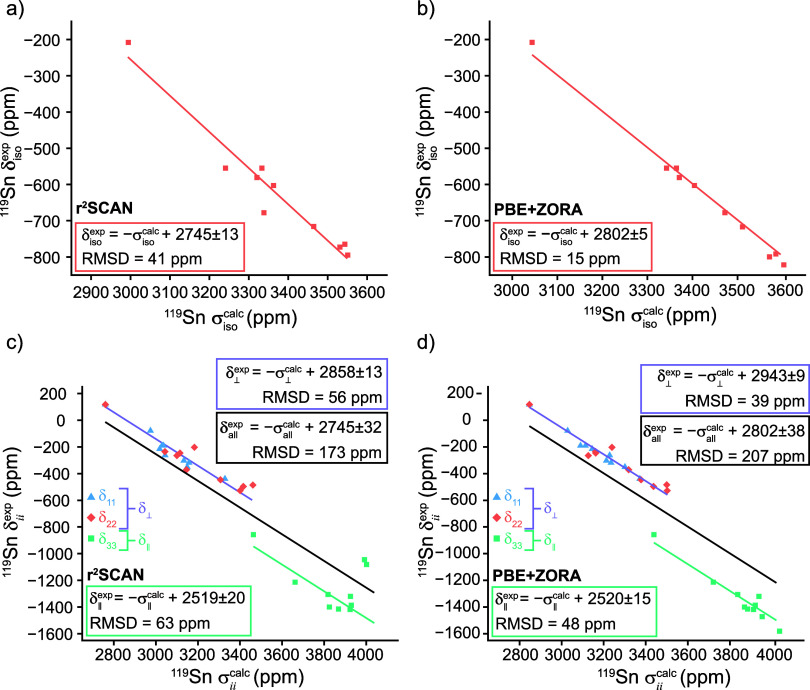
Plot of experimental ^119^Sn chemical shifts against calculated magnetic shieldings for Sn­(II) MOFs and SnO obtained from (a, c) nonrelativistic (r^2^SCAN) and (b, d) scalar-relativistic (PBE+ZORA) DFT calculations. (a, b) Isotropic shifts (δ_iso_^exp^) and shieldings (σ_iso_^calc^). (c, d) Principal components (δ_*ii*_^calc^ and σ_*ii*_^calc^). Linear fits with a fixed slope of −1 are shown together with the corresponding fit parameters.

^119^Sn–^117^Sn *J* couplings were also calculated for Sn_2_(DOBDC) and Li_2_Sn_2_(1,4-BDC)_3_(H_2_O)_2_ using PBE+ZORA, yielding values that are in reasonable agreement (within 15%) of those measured experimentally (Tables S6 and S7). This shows that these DFT calculations are capable of capturing *J* coupling interactions between moderately heavy Sn atoms, both through-bond and through-space.^62^

Having established the good agreement of the calculated isotropic chemical shifts, the CSA was considered. The calculated spans correlate linearly with the experimental values but are all underestimated by a constant value of ∼300 and ∼400 ppm for r^2^SCAN and PBE+ZORA, respectively (Figure 9a,b). To understand this discrepancy, the experimental shift tensor components were plotted against the calculated shielding tensor components from r^2^SCAN and PBE+ZORA (Figure 8c,d). In both cases, σ_11_^calc^ and σ_22_^calc^ consistently lie above the regression from the isotropic values (Figure 8a,b), whereas σ_33_^calc^ falls below it, separating the components into two distinct regimes. This explains the discrepancy between the experimental and calculated CSAs (Figure 9a,b), but is masked by the isotropic averaging in Figure 8a,b. Notably, this effect is present for both r^2^SCAN and PBE+ZORA with similar magnitudes, suggesting that zeroth-order relativistic effects are not the underlying cause.

**Figure 9:**
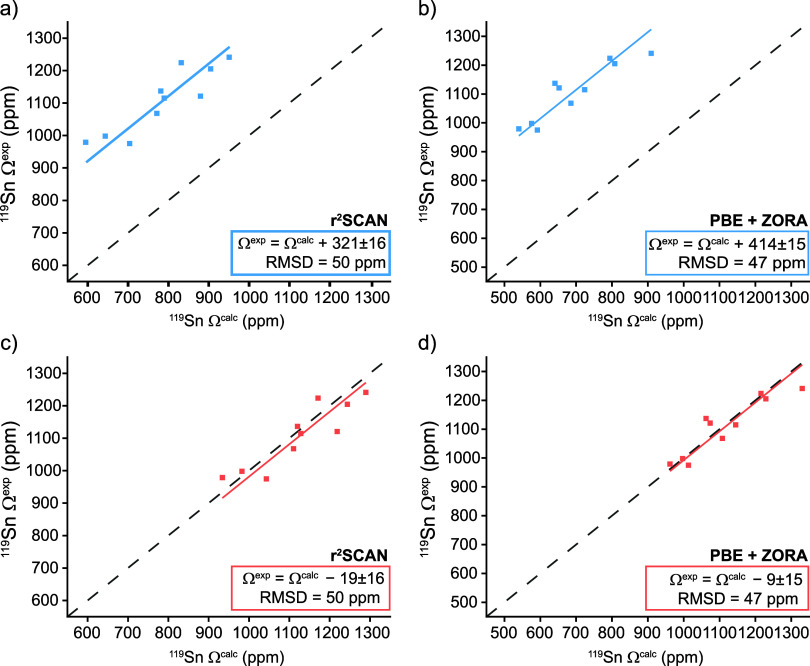
Correlation between calculated and experimental ^119^Sn spans (Ω^calc^ vs Ω^exp^) from nonrelativistic (r^2^SCAN) and scalar-relativistic (PBE+ZORA) DFT calculations. Panels (a, b) present Ω^calc^ values referenced using a single reference shielding (σ_ref_) derived from the linear regression of calculated isotropic shieldings against experimental isotropic shifts (σ_iso_^calc^ vs δ_iso_^exp^; Figure 8a,b and Table 3). Panels (c, d) show analogous plots in which Ω^calc^ values were obtained using two reference shieldings for the directions parallel and perpendicular to the lone pair, i.e., σ_∥_^calc^ vs δ_∥_^exp^ and σ_⊥_^calc^ vs δ_⊥_^exp^ (Figure 8c,d and Table 3). Solid lines are linear regressions with a fixed gradient of 1. Dashed lines show Ω^calc^ = Ω^exp^.

In order to restore the predictive power of DFT calculations for CSAs, we fitted the parallel and perpendicular tensor components with separate linear regressions, constraining the slope to −1, to give distinct reference shieldings, σ_∥_^ref^ and σ_⊥_^ref^ (Figure 8c,d and Table 3). This markedly improves the fit as compared to a linear regression of all principal components. The calculated chemical shift tensor components obtained from the two regressions yield calculated CSAs with excellent quantitative agreement with experiment, correcting the offset (Figure 9c,d). This demonstrates that component-specific referencing effectively captures the key anisotropic effects of Sn­(II), and the approach is justified below.

**Table 3:** Statistical Parameters Obtained from Linear Regression (Fixed Slope of −1) of Experimental ^119^Sn Chemical Shifts against Calculated Magnetic Shieldings from Nonrelativistic (r^2^SCAN) and Scalar-Relativistic (PBE+ZORA) Calculations^a^

	r^2^SCAN			PBE+ZORA		
Fitted Parameter	σ_ref_ (ppm)	*R* ^2^	RMSD (ppm)	σ_ref_ (ppm)	*R* ^2^	RMSD (ppm)
σ_iso_^calc^	2745 ± 13	0.94	41.0	2802 ± 5	0.99	14.7
σ_all_^calc^	2745 ± 32	0.90	172.7	2802 ± 38	0.85	206.8
σ_⊥_^calc^	2858 ± 13	0.90	56.2	2943 ± 9	0.95	39.0
σ_∥_^calc^	2519 ± 20	0.89	62.7	2520 ± 15	0.93	48.4

a Reported quantities include the coefficient of determination (*R* ^2^), root-mean-square deviation (RMSD), and fitted reference shieldings (σ_ref_). Fits were performed for the calculated isotropic shieldings (σ_iso_^calc^), all shielding tensor principal components together (σ_all_^calc^), and for principal components orientated parallel and perpendicular to the Sn­(II) lone pair (σ_∥_^calc^ and σ_⊥_^calc^).

An alternative approach to correct for discrepancies between calculated shieldings and experimental shifts has been to apply a scaling factor (i.e., δ^calc^ = σ^ref^ – *m*σ^calc^, where *m* ≠ 1).^30,61,63–67^ However, for the systems studied here, this method yields significantly poorer agreement between experimental and calculated CSAs (Figure S10).

The two-regression spans calculated with PBE+ZORA have slightly better agreement with experiment than those from r^2^SCAN (RMSD = 47.3 vs 49.6 ppm). This shows that inclusion of relativistic effects marginally improves predictive power, but accounting for the stereoactive Sn­(II) lone pair is a more important factor. The calculated skews improve slightly using the two regressions (Figure S11); however, there is still significant error since the skew depends on the difference between tensor components, compounding the influence of uncertainties in the individual principal components, for both experimental and calculated data sets.^34^

To relate the CSA tensor to the local Sn structure, the electron localization function (ELF) map was calculated using DFT, and a visualization of the calculated magnetic shielding tensor overlaid (Figure 10). This demonstrates that the principal axis of the CSA tensor (σ_33_) is aligned with the resolved stereoactive Sn­(II) lone pair. The orientation of the stereoactive Sn­(II) lone pair, along σ_33_^calc^, allows the systematic difference between the calculated principal components to be understood: it appears that the lone pair strongly perturbs σ_33_^calc^ relative to σ_11_^calc^ and σ_22_^calc^, justifying the use of two regressions. Taken together, it can be seen that the ^119^Sn CSA depends strongly on the stereoactive lone pair and, in combination with appropriately calibrated DFT calculations, ^119^Sn NMR can therefore precisely probe the geometry of the lone pair.

**Figure 10:**
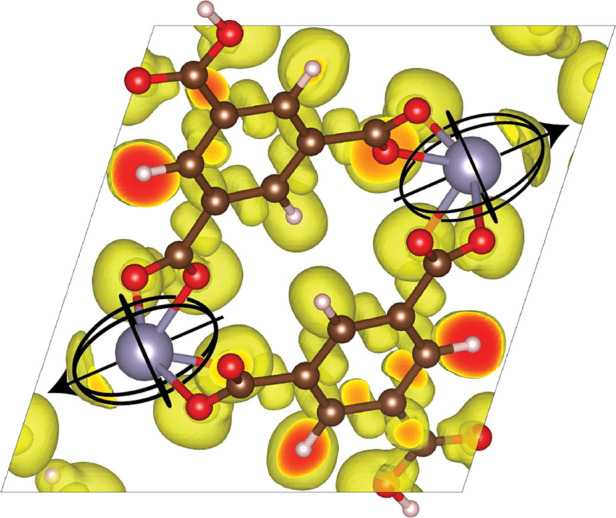
Electron localization function (ELF) map of Sn­(H-1,3,5-BTC) showing ^119^Sn lone pairs with calculated magnetic shielding tensors overlaid in black. The arrows indicate the σ_33_ principal components, oriented toward the lone pairs.

Finally, we consider the reported application of Sn­(II) MOFs as lithium battery anodes to examine whether ^119^Sn NMR has the potential to provide mechanistic information on charge storage in these materials. Liu et al. put forward a mechanism for lithiation of Sn_2_(DOBPDC) involving Li^+^ insertion, amorphization of the MOF, and the formation of a Li–Sn alloy.^10^ ^119^Sn NMR could provide further insights into this process, and as a demonstration, we performed preliminary calculations following addition of a Li^+^ ion to Sn­(H-1,2,4-BTC) (Figure S12). Sn sites adjacent to the intercalated lithium ion show a marked change in the ^119^Sn isotropic shift and CSA; in particular, the increased ^119^Sn CSA is strong evidence of interaction between the Sn­(II) lone pair and the Li^+^ cation. This site-specific insight paves the way for mechanistic studies of Li^+^ intercalation, and host–guest chemistry in general, in Sn­(II) MOFs.

## Conclusions

By combining variable MAS and static ultra-wideline BRAIN-CP-WCPMG experiments, the ^119^Sn CSA parameters have been measured for 9 sites in 6 MOFs that contain Sn­(II) metal nodes. The stereoactive Sn­(II) lone pair was found to give large ^119^Sn CSAs with spans ranging from 979 to 1241 ppm. ^119^Sn–^115/117^Sn *J* coupling was observed in two MOFs with magnitudes of 10.6 and 4.8 kHz, corresponding to through-bond and apparently through-space interactions, when there is overlap of the lone pairs.

Using the experimental data, we have developed an approach to accurately calculate ^119^Sn isotropic chemical shifts and CSAs using DFT. While a single shift–shielding regression yields accurate isotropic shifts, it fails to reproduce the CSA. Component-level analysis reveals systematic differences between calculated shielding tensor components, arising from the stereoactive Sn­(II) lone pair. Using two different regressions parallel and perpendicular to the lone pair eliminates systematic errors in calculated spans and yields near-quantitative agreement with experimental results. Specifically, using PBE+ZORA, we obtain root-mean squared deviations of 14.7 and 47.3 ppm for the isotropic ^119^Sn shifts and spans, respectively. This work shows how large chemical shift anisotropies can be measured experimentally and accurately calculated, even for heavier elements.

The combination of ^119^Sn NMR and DFT sheds light on the Sn­(II) lone pair. The geometry of the lone pair can be determined with exceptional sensitivity and this method can be used to probe the interaction between intercalated species and the lone pair, for example following lithiation in a Li-ion battery,^7,10–13^ where the mechanism of electrochemical charge storage has not yet been fully elaborated on the local scale. In general, harnessing anisotropic NMR enables atomic-scale structural determination of complex materials, such as Sn­(II) MOFs, but will be applicable to many other types of material that contain these cations, such as halide perovskites, oxides, and phosphates.

## Experimental Section

### Synthesis

(Sn­(H-1,2,4-BTC), Sn­(H-1,3,5-BTC), Sn_2_(1,3,5-BTC)­(OH)), and Sn_2_(DOBDC) were synthesized according to the procedure of Ramana et al.^8^ Li_2_Sn_2_(BDC)_3_(H_2_O)_2_ was synthesized according to the procedure of Wang et al.,^37^ but altering the hydrothermal crystallization period from 6 days to 3 days. Sn_3_O­(1,4-BDC)_2_ was synthesized by modifying the procedure of Wang et al., replacing TMA_2_(1,4-BDC) with KOH and H_2_-1,4-BDC.^38^ The chemicals were used as provided from various suppliers: SnSO_4_ (Acros Organics, 97%), KOH (Fisher Scientific, >85%), H_3_-1,2,4-BTC (Fisher Scientific, 98%), H_3_-1,3,5-BTC (Alfar-Aesar, 98%), H_4_-DOBDC (ABCR, 97%), and H_2_-1,4-BDC (Alfar-Aesar, (98+%).

**Sn­(H-1,2,4-BTC)** was prepared by adding SnSO_4_ (0.35 g, 1.63 mmol), KOH (0.12 g, 2.14 mmol), and H_3_-1,2,4-BTC (0.58 g, 2.76 mmol) to deionized water (6 mL) in a 20 mL Teflon autoclave liner. The mixture was then stirred for 45 min and then placed in an autoclave for 72 h at 170 °C.

**Sn­(H-1,3,5-BTC)** was prepared by adding SnSO_4_ (0.32 g, 1.49 mmol), KOH (0.12 g, 2.14 mmol), and H_3_-1,3,5-BTC (0.58 g, 2.76 mmol) to deionized water (6 mL) in a 20 mL Teflon autoclave liner. The mixture was then stirred for 45 min and then placed in an autoclave for 72 h at 170 °C.

**Sn** **_2_** **(DOBDC)** was prepared by adding SnSO_4_ (0.10 g, 0.47 mmol), KOH (0.03 g, 0.53 mmol), and H_4_-DOBDC (0.06 g, 0.30 mmol) to deionized water (6 mL) in a 20 mL Teflon autoclave liner. The mixture was then stirred for 60 min and then placed in an autoclave for 60 h at 130 °C.

**Sn** **_2_** **(H-1,3,5-BTC)­(OH)** was prepared by adding SnSO_4_ (0.10 g, 0.47 mmol), LiOH·H_2_O (0.031 g, 0.75 mmol), and H_3_-1,3,5-BTC (0.58 g, 2.76 mmol) to deionized water (2 mL) and methanol (4 mL) in a 20 mL Teflon autoclave liner. The mixture was then stirred for 45 min and then placed in an autoclave for 72 h at 170 °C.

**Li** **_2_** **Sn** **_2_** **(BDC)** **_3_** **(H** **_2_** **O)** **_2_** was prepared by adding SnSO_4_ (2.15 g, 10.0 mmol), LiOH·H_2_O (0.98 g, 23.6 mmol), and H_2_-1,4-BDC (0.58 g, 3.49 mmol) to deionized water (2 mL) and methanol (4 mL) in a 20 mL Teflon autoclave liner. The mixture was then stirred for 45 min and then placed in an autoclave for 72 h at 170 °C.

**Sn** **_3_** **O­(1,4-BDC)** **_2_** was prepared by adding SnSO_4_ (0.33 g, 1.54 mmol), KOH (0.13 g, 2.32 mmol), and H_2_-1,4-BDC (0.58 g, 3.49 mmol) to deionized water (6 mL) in a 20 mL Teflon autoclave liner. The mixture was then stirred for 45 min and then placed in an autoclave for 72 h at 170 °C.

### Materials Characterization

#### Powder X-ray Diffraction

The powder XRD data were collected using a Siemens D5000 X-ray diffractometer using Cu Kα_1/2_ radiation (λ = 1.5418 Å) at room temperature. Theoretical powder diffraction patterns for each MOF were generated using VESTA^68^ based on the corresponding crystallographic information files.

#### Solid-State NMR Experiments

Samples were packed into 3.2 mm outer-diameter zirconia MAS rotors. Solid-state NMR measurements were either taken on a Bruker Avance Neo 500 MHz (11.7 T) or a Bruker Avance III 500 MHz (11.7 T) spectrometer. The frequencies of the studied nuclei are ^1^H 500.2 and ^119^Sn 186.4 MHz. The experiments were performed on either a 3.2 mm double resonance or triple resonance MAS probe, at spinning speeds from 10–18 kHz. All experiments were run at room temperature. The ^119^Sn spectra were referenced to the shift of tetracyclohexyltin at −97.3 ppm. All spectra were recorded using proton decoupling power levels of ω_1_(^1^H)/2π = 50–80 kHz. Saturation recovery experiments were run on each sample to determine the ^1^H and ^119^Sn *T*_1_ constants (Table S1). In the ^119^Sn *T*_1_ experiments multiple peaks were integrated over and an average *T*_1_ was recorded to increase accuracy in the measurement. The ^119^Sn *T*_1_ for Sn_2_(1,3,5-BTC)­(OH) was measured using a static BRAIN-CP *T*_1_ experiment,^69^ so the measured value is an average of the two Sn environments in the sample.

MAS ^119^Sn NMR spectra were recorded with a single-pulse experiment at two or more different spinning speeds to confirm the position of the isotropic resonance. The pulse length was chosen to be sufficiently short to excite the whole spectrum. The recycle delay was chosen based on the measured *T*_1_ and the pulse length to give the optimal signal-to-noise ratio, using the Ernst angle formula (Table S2).^70,71^ The single-pulse data was processed by right-shifting by the dead-time (12–20 points) to give the correct time zero and back predicting the first 20–120 points (including the dead time) to remove the effects of probe ringing. The MAS spectra of Sn_2_(1,3,5–BTC)­(OH) was recorded with a WURST-CPMG experiment with the parameters in Table S3. A MAS ^119^Sn NMR spectrum of H-1,2,4-BTC was recorded using a BRAIN-CP-WCPMG MAS experiment with the parameters in Table S5. Static ^119^Sn NMR spectra were recorded using a BRAIN-CP-WCPMG experiment with the parameters in Table S4 and a recycle delay equal to 1.3× the ^1^H *T*_1_ (Table S1).

The spinning sidebands were fit to find the CSA parameters using the CSA fitting program of ssNake.^39^ CSA parameters are reported using the Herzfeld–Berger convention, where the principal components of the chemical shift tensor are ordered such that

$$\delta_{11}\geq \delta_{22}\geq \delta_{33}$$ 2

The isotropic chemical shift, δ_iso_, is then defined as the average of the principal components

$$\delta_{\mathrm{iso}}=\frac{\delta_{11}+\delta_{22}+\delta_{33}}{3}$$ 3

The span, Ω, describes the magnitude of the CSA in ppm:

$$\Omega =\delta_{11}-\delta_{33}$$ 4

and the skew, κ, is a dimensionless parameter that describes the deviation from axial symmetry:

$$\kappa =\frac{3\left(\delta_{22}-\delta_{\mathrm{iso}}\right)}{\Omega }$$ 5

Numerical NMR simulations were performed in SIMPSON.^72^

#### Calculation of NMR Parameters

DFT calculations were performed using the CASTEP plane-wave code (v25.1.2). Initial atomic coordinates were taken from experimentally determined crystal structures,^8,37,38^ and their geometries were optimized prior to the calculation of NMR parameters. Two sets of calculations were performed: (1) Nonrelativistic calculations were performed using the regularized-restored strongly constrained and appropriately normed (r^2^SCAN) meta-generalized gradient approximation (meta-GGA) functional, and (2) Relativistic calculations were performed using the Perdew–Burke–Ernzerhof (PBE) GGA functional with the zeroth-order regular approximation (ZORA). Core–valence interactions were described using ultrasoft pseudopotentials.^73^ Geometry optimizations were performed with a plane-wave cutoff energy of 500 eV, and the Brillouin zone was sampled using a Monkhorst–Pack *k*-point grid^74^ with a *k*-point spacing of 0.08 Å^–1^ in reciprocal space. Both internal atomic coordinates and lattice parameters were allowed to relax, with convergence tolerances of 0.05 eV Å^–1^ for forces, 2 × 10^–5^ eV for total energy, and 2 × 10^–3^ Å for atomic displacements. For Sn­(II) MOFs, NMR parameters were calculated using a plane-wave cutoff of 900 eV and a *k*-point spacing of 0.06 Å^–1^, while for SnO a cutoff of 1000 eV and a *k*-point spacing of 0.03 Å^–1^ were employed. All calculations were carried out on the Avon HPC cluster at the Scientific Computing Research Technology Platform, University of Warwick, and the Young compute cluster at University College London.

## Supplementary Material



## Data Availability

Raw and processed NMR data, XRD patterns, and DFT input and output files are available at DOI: 10.5281/zenodo.19367933.

## Notes

Experimental NMR parameters, XRD data, SIMPSON simulations, additional figures of the MOF structures, and supplementary data figures (PDF)
