# Supplementary material for: Combining Anisotropic 119Sn NMR and Ab Initio Calculations to Probe the Stereoactive Lone Pair in Sn(II) Metal–Organic Frameworks
Source: Chem Mater. 2026 Jun 15;38(13):6395–406. doi: 10.1021/acs.chemmater.6c00369 (PMC13374023; doi:10.1021/acs.chemmater.6c00369)
Supplement: Supplementary file 1 [file cm6c00369_si_001.pdf]

Supporting Information

Combining Anisotropic ^{119}Sn NMR and Ab Initio Calculations to Probe the Stereoactive Lone Pair in Sn(II) Metal–Organic Frameworks

Helena R. Loan[a], Ryan J. Bragg[a], Caitlyn F. Walton[a], Richard I. Walton[a,*], Michael A. Hope[a,*]

[a]Department of Chemistry, University of Warwick, Coventry, CV4 7AL

*R.I.Walton@warwick.ac.uk, Michael.Hope.1@warwick.ac.uk

Table S1: T_1 relaxation data for the ^{119}Sn and ^{1}H nuclei of the different MOFs

	^{119}Sn T_1 (s)	^{1}H T_1 (s)
Sn(H-1,2,4-BTC)	68	3.9
Sn(H-1,3,5-BTC)	54	1.8, 69.5
Sn_2(DOBDC)	173	17.2
$Li_2Sn_2(1,4\text{-}BDC)_3(H_2O)_2$	175	2.4
Sn_2(1,3,5-BTC)(OH)	119	3.6
$Sn_3O(1,4\text{-}BDC)_2$	-	190.7

Table S2: Experimental Parameters for ^{119}Sn MAS one-pulse experiments

	Pulse length (µs)	Pulse power (kHz)	Flip Angle (°)	Recycle Delay (s)
Sn(H-1,2,4-BTC)	2.0	125.0	90.0	60
Sn(H-1,3,5-BTC)	2.0	63.7	45.9	60
Sn_2(DOBDC)	2.0	81.3	58.5	104
$Li_2Sn_2(1,4\text{-}BDC)_3(H_2O)_2$	2.0	87.0	62.6	60
$Sn_3O(1,4\text{-}BDC)_2$	3.1	80.6	90.0	180

Table S3: Experimental Parameters for ^{119}Sn WURST-QCPMG Experiments

Number of Transients	144
Recycle Delay	150
Spectral Window Width (kHz)	1000
Number of Meiboom-Gill Loops (N)	20
Spin Echo Length (µs)	1928
Acquisition Time (ms)	41.0
WURST Length (µs)	50
WURST Sweep width (kHz)	500
WURST Amplitude (kHz)	40
Sweep Direction	Low to high

Table S4: Experimental Parameters for ^{119}Sn BRAIN-CP-WCPMG experiments

Number of Transients	256 – 5400
Recycle Delays (s)	3 – 250
Spectral Window Width (kHz)	1000
Number of Meiboom-Gill Loops (N)	100 – 200
Spin Echo Length (μs)	125 – 300
Acquisition Time (ms)	40.1
^{1}H Excitation Pulse Length (μs)	2.5
^{1}H Excitation Pulse Amplitude (kHz)	100
^{1}H CP Pulse Amplitude (kHz)	50
CP Contact Time (ms)	8
CP-WURST Amplitude (kHz)	50
CP-WURST Sweep Width (kHz)	250 – 400
Refocusing WURST Length (μs)	50
Refocusing WURST Sweep Width (kHz)	500
Refocusing WURST Amplitude (kHz)	40
Sweep Direction	Low to high

Table S5: Experimental Parameters for ^{119}Sn BRAIN-CP-WCPMG MAS experiment

Spinning Speed	12.5
Number of Transients	128
Recycle Delays (s)	8
Spectral Window Width (kHz)	1000
Number of Meiboom-Gill Loops (N)	50
Spin Echo Length (μs)	890
Acquisition Time (ms)	48.5
^{1}H Excitation Pulse Length (μs)	2.5
^{1}H Excitation Pulse Amplitude (kHz)	101
^{1}H CP Pulse Amplitude (kHz)	19
CP Contact Time (ms)	8
CP-WURST Amplitude (kHz)	10
CP-WURST Sweep Width (kHz)	12.5
Refocusing WURST Length (μs)	50
Refocusing WURST Sweep Width (kHz)	500
Refocusing WURST Amplitude (kHz)	26
Sweep Direction	Low to high

Table S6: Calculated (PBE+ZORA) ^{119}Sn–^{117}Sn J couplings for $Li_2Sn_2(1,4\text{-}BDC)_3(H_2O)_2$. The average calculated J coupling across all sites is 5.3 kHz with a standard deviation of 0.9 kHz which is in good agreement with the experimentally measured 4.8 kHz.

Perturbed ^{119}Sn site	Coupled ^{117}Sn site	^{119}Sn–^{117}Sn J Coupling (kHz)
Sn 1	Sn 2	4.9
Sn 1	Sn 3	3.9
Sn 1	Sn 4	6.9
Sn 2	Sn 1	4.9
Sn 2	Sn 3	6.0
Sn 2	Sn 4	5.9
Sn 3	Sn 1	3.9
Sn 3	Sn 2	6.0
Sn 3	Sn 4	4.9
Sn 4	Sn 1	6.9
Sn 4	Sn 2	5.9
Sn 4	Sn 3	4.9
Sn 5	Sn 8	6.7
Sn 5	Sn 6	4.1
Sn 5	Sn 7	4.9
Sn 6	Sn 8	5.1
Sn 6	Sn 5	4.9
Sn 6	Sn 7	6.2
Sn 7	Sn 6	6.2
Sn 7	Sn 8	5.4
Sn 7	Sn 5	4.1
Sn 8	Sn 6	5.1
Sn 8	Sn 5	6.7
Sn 8	Sn 7	5.4

Table S7: Experimental J couplings and calculated (PBE+ZORA) ^{119}Sn–^{117}Sn J couplings for $Sn_2(DOBDC)$ and $Li_2Sn_2(1,4\text{-}BDC)_3(H_2O)_2$. The average calculated J coupling value across all sites, along with its standard deviation, is presented for $Li_2Sn_2(1,4\text{-}BDC)_3(H_2O)_2$.

	Experimental J Coupling (kHz)	Calculated ^{119}Sn–^{117}Sn J Coupling (kHz)
$Sn_2(DOBDC)$	10.7	12.1
$Li_2Sn_2(1,4\text{-}BDC)_3(H_2O)_2$	4.8	5.3 ± 0.9

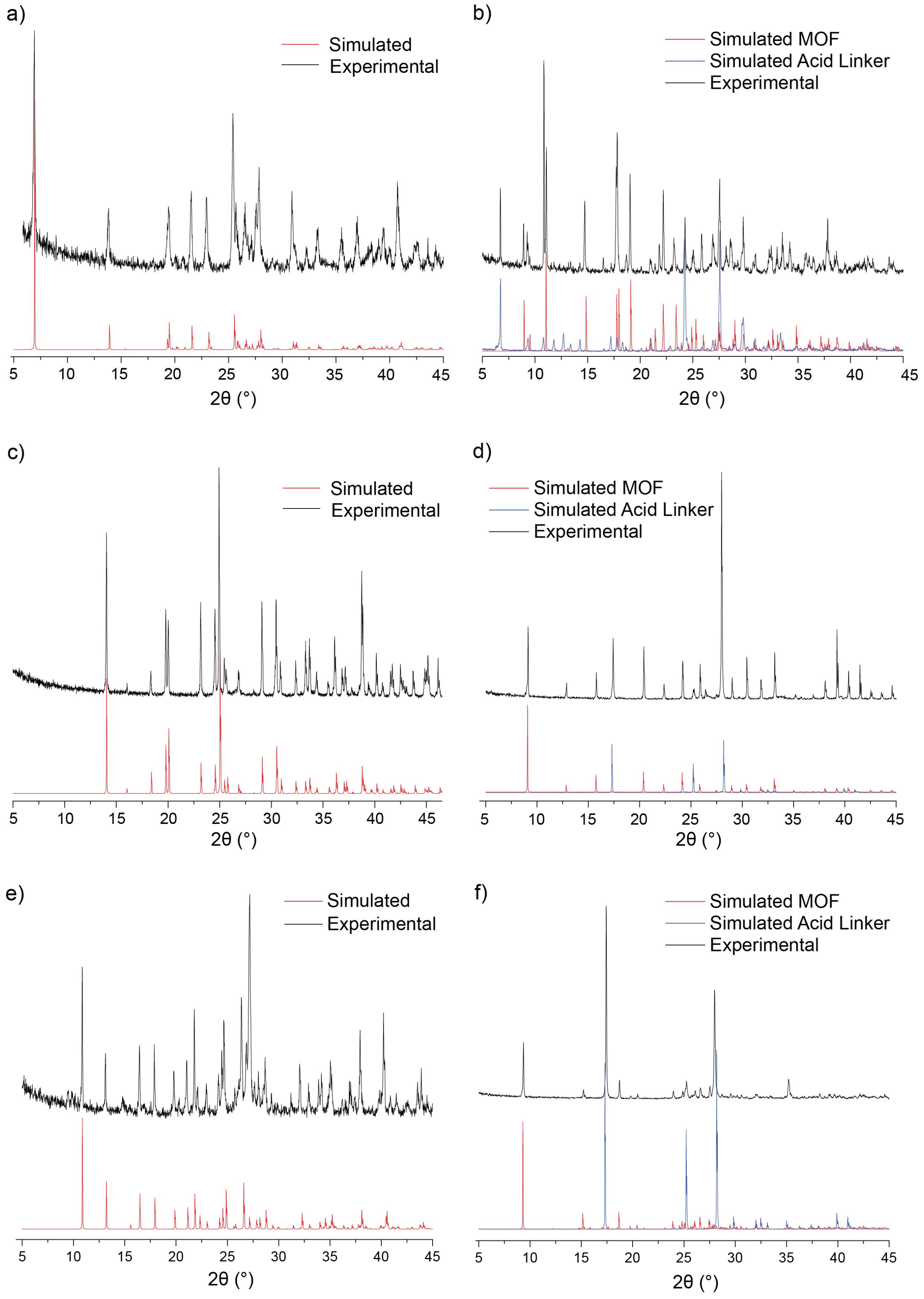

Figure S1: Experimental and simulated PXRD data of (a) Sn(H-1,2,4-BTC), (b) Sn(H-1,3,5-BTC), (c) Sn_2(DOBDC), (d) $Li_2Sn_2(1,4\text{-}BDC)_3(H_2O)_2$, (e) Sn_2(1,3,5-BTC)(OH), and (f) $Sn_3O(1,4\text{-}BDC)_2$.

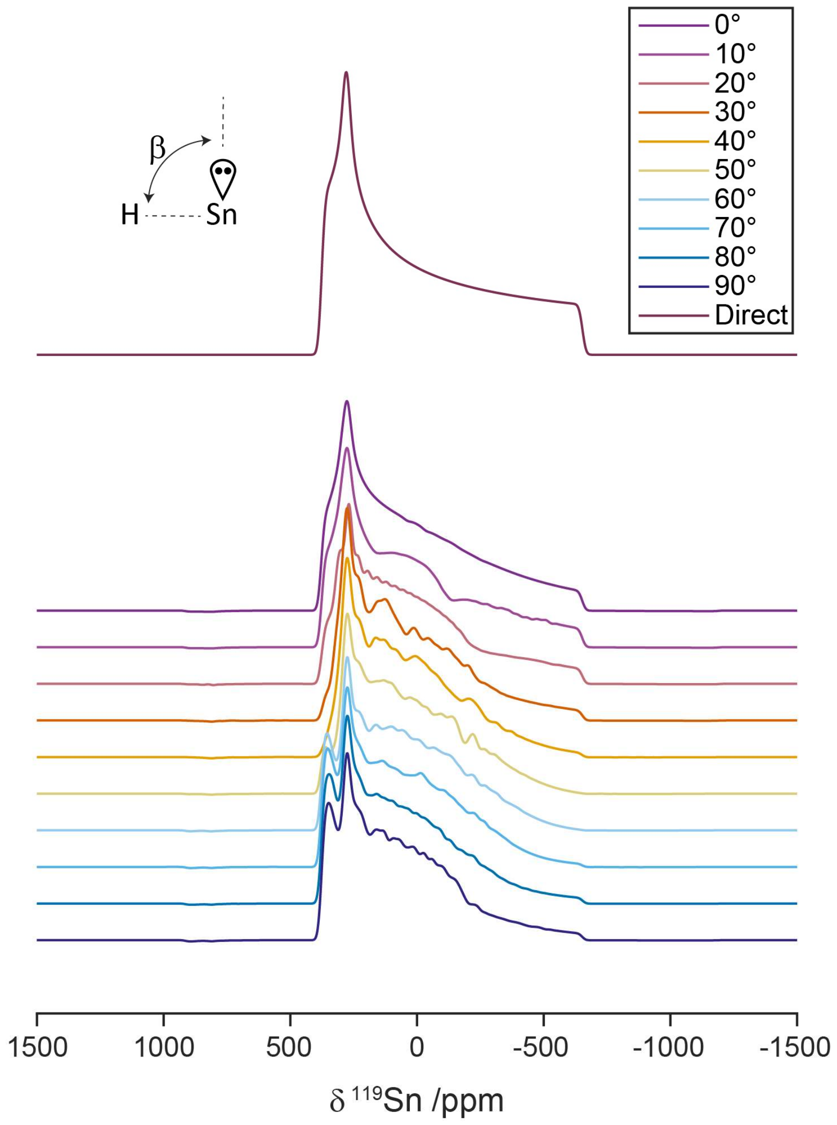

Figure S2: SIMPSON simulations of static ^{119}Sn spectra with Ω = 1024 ppm, κ = 0.81 at 11.7 T. Top: direct excitation. Bottom: ^{1}H→^{119}Sn CP with ν(^{1}H) = ν(^{119}Sn) = 1 MHz and a contact time of 8 ms, as a function of the β Euler angle between the principal axes of the Sn CSA tensor (i.e., the direction of the lone pair) and the ^{1}H–^{119}Sn dipolar tensor. The α and γ Euler angles are zero. The zcw28656 crystal file was used, 100 kHz of continuous-wave ^{1}H decoupling was applied during acquisition, and 5000 Hz of gaussian line-broadening. There is a notable decrease in the intensity at the low frequency side with increasing β, especially when β is close to the magic angle of 54.7°. This is because isochromats with low frequencies correspond to crystallites where the CSA tensor (Sn lone pair) is aligned with B_0; as a result, the β angle is also the angle between the ^{1}H–^{119}Sn dipolar tensor and B_0 for these crystallites, and the dipolar coupling is zero when this is equal to the magic angle. The closest protons in the various MOF structures are typically >45° away from the direction of the Sn lone pair, which could explain the drop of intensity at lower frequency observed experimentally, although the effect would be less pronounced with multiple ^{1}H present. In summary, the efficiency of CP is orientation dependent, modulating the intensity of an observed CSA powder pattern. Note that the simulations here are for conventional CP, but the phenomenon is expected to also apply to the BRAIN-CP experiments used in the main text.

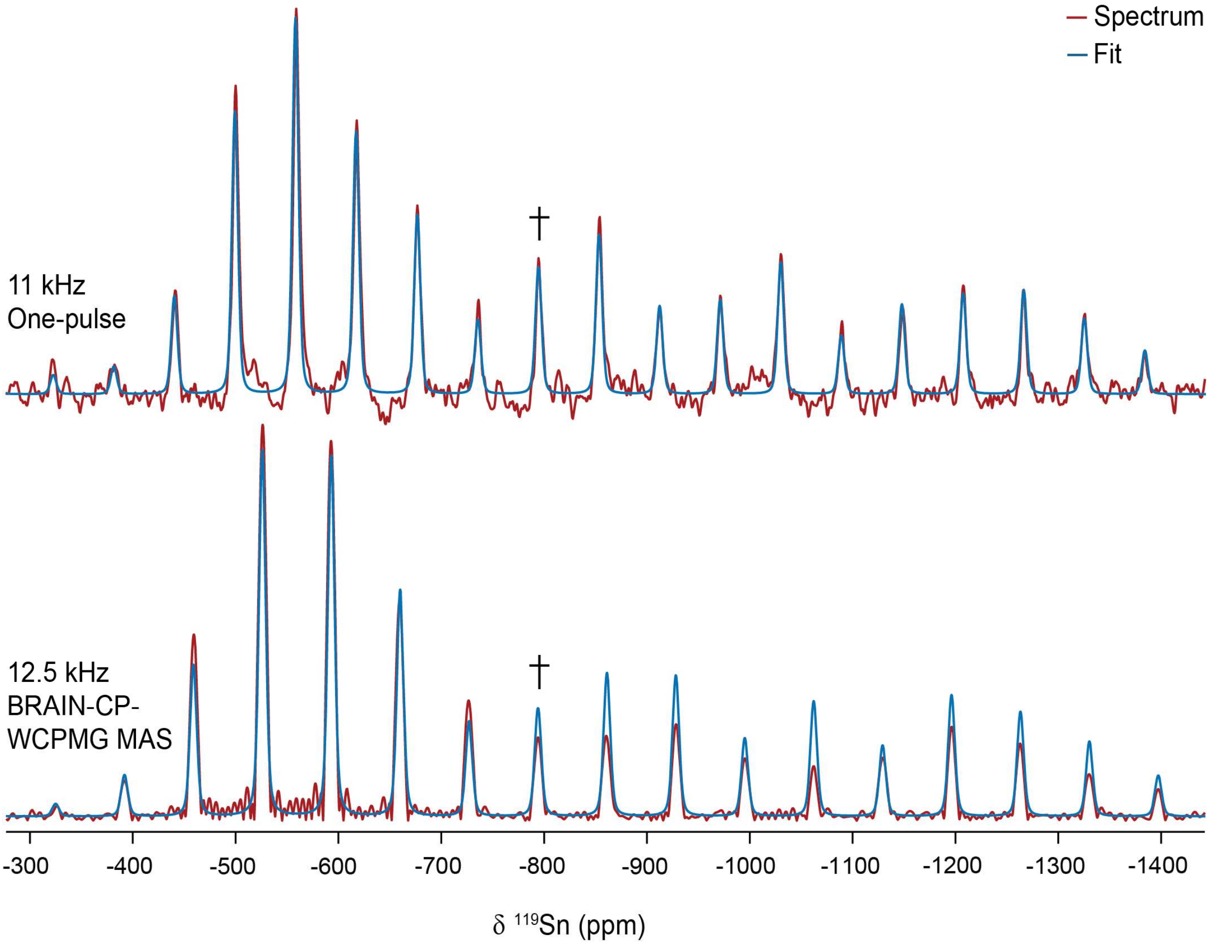

Figure S3: ^{119}Sn NMR spectra (11.7 T) and fitted spectra of Sn(H-1,2,4-BTC) with δ_{iso} = −795 ppm, Ω = 979 ppm and κ = 0.82. The isotropic shift is given by †. Top: 11 kHz MAS using a one pulse sequence. Bottom: 12.5 kHz using a BRAIN-CP-WCPMG MAS pulse sequence (experimental parameters in **Table S5**). The second spectrum has 11 times greater sensitivity (SNR per √time) due to the greater magnetisation of ^{1}H nuclei and the shorter T_1 allows for shorter acquisition times (17 min compared to 7.5 h). To allow more echoes to be measured and improve the SNR, a spin echo length of 12 rotor periods was used, which causes low intensity truncation artefacts. Although the sensitivity of the BRAIN spectrum is higher, there are discrepancies with the fitted spectrum, which would need to be addressed or accounted for.

Sn(H-1,3,5-BTC)

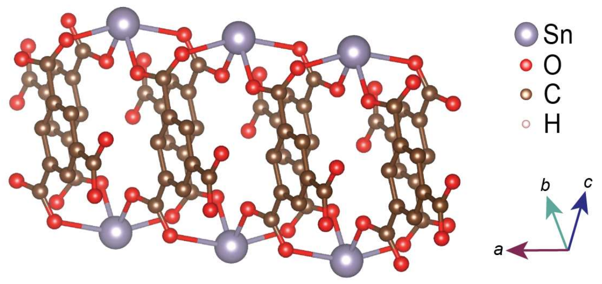

Figure S4: View of Sn(H-1,3,5-BTC) forming tubular chains in the *a* axis.[8]

Sn_2DOBDC

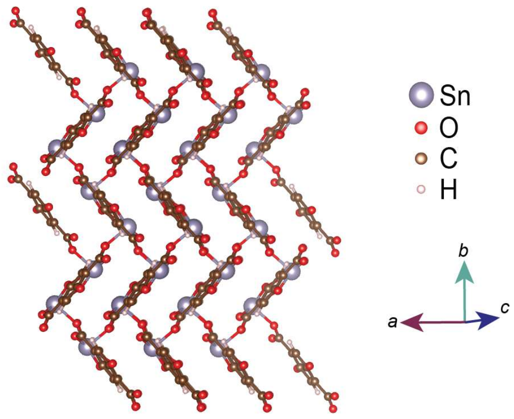

Figure S5: Structure of Sn_2DOBDC showing the herringbone arrangement of the dimeric units. [8]

a) $Li_2Sn_2(1,4\text{-}BDC)_3(H_2O)_2$ b)

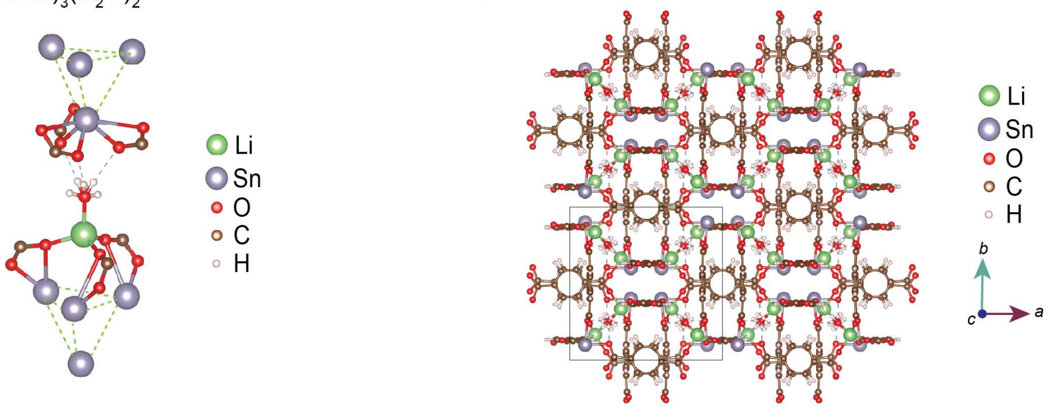

Figure S6: Structures of $Li_2Sn_2(1,4\text{-}BDC)_3(H_2O)_2$[37] showing a) coordination of Sn_4 tetrahedra with Li and water and b) an ordered 3D framework viewed along the *c* axis.

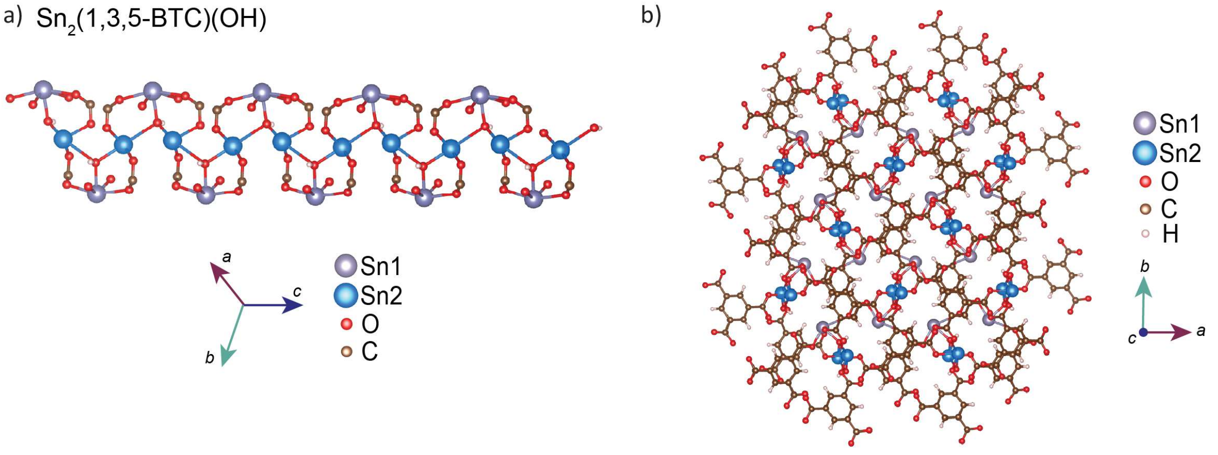

Figure S7: Sn_2(1,3,5-BTC)(OH).[8] a) Infinite inorganic chains of Sn2 connected via a bridging hydroxide. b) Viewed along the *c* axis, demonstrating narrow voids.

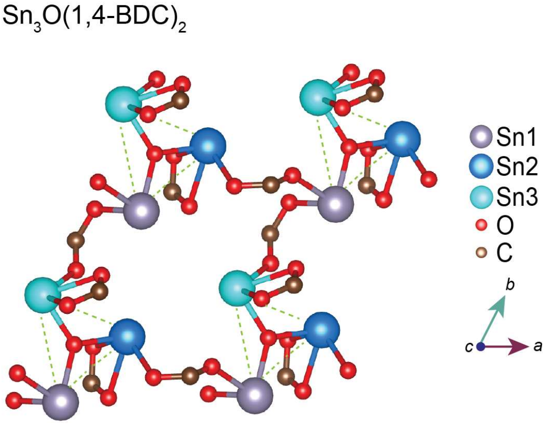

Figure S8: Structure of Sn_3O(1,4-BDC)$_2$ showing bonding of adjacent Sn_3O trimers via carboxylate groups on the ligand to form layers in the *ab* plane.[38]

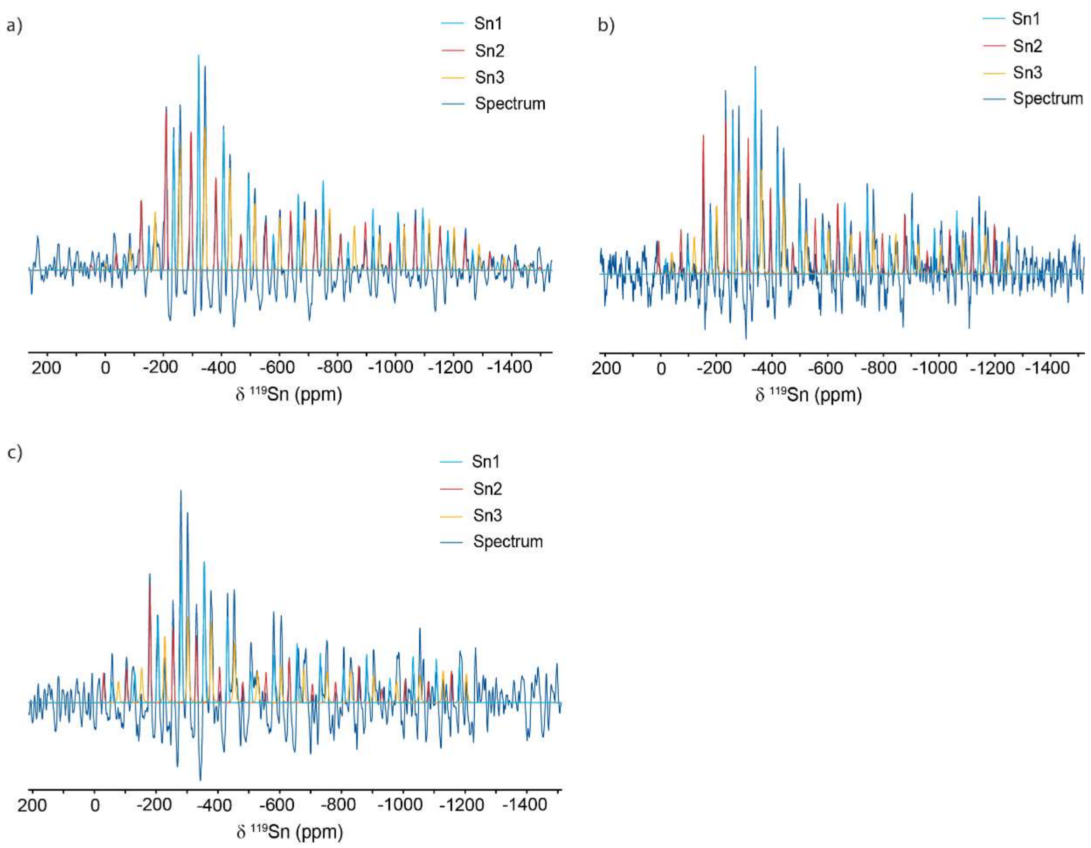

Figure S9: Simultaneously fitted ^{119}Sn NMR spectra of $Sn_3O(1,4\text{-}BDC)_2$ using a one-pulse sequence at MAS rates of a) 16 kHz, b) 15 kHz, and c) 14 kHz.

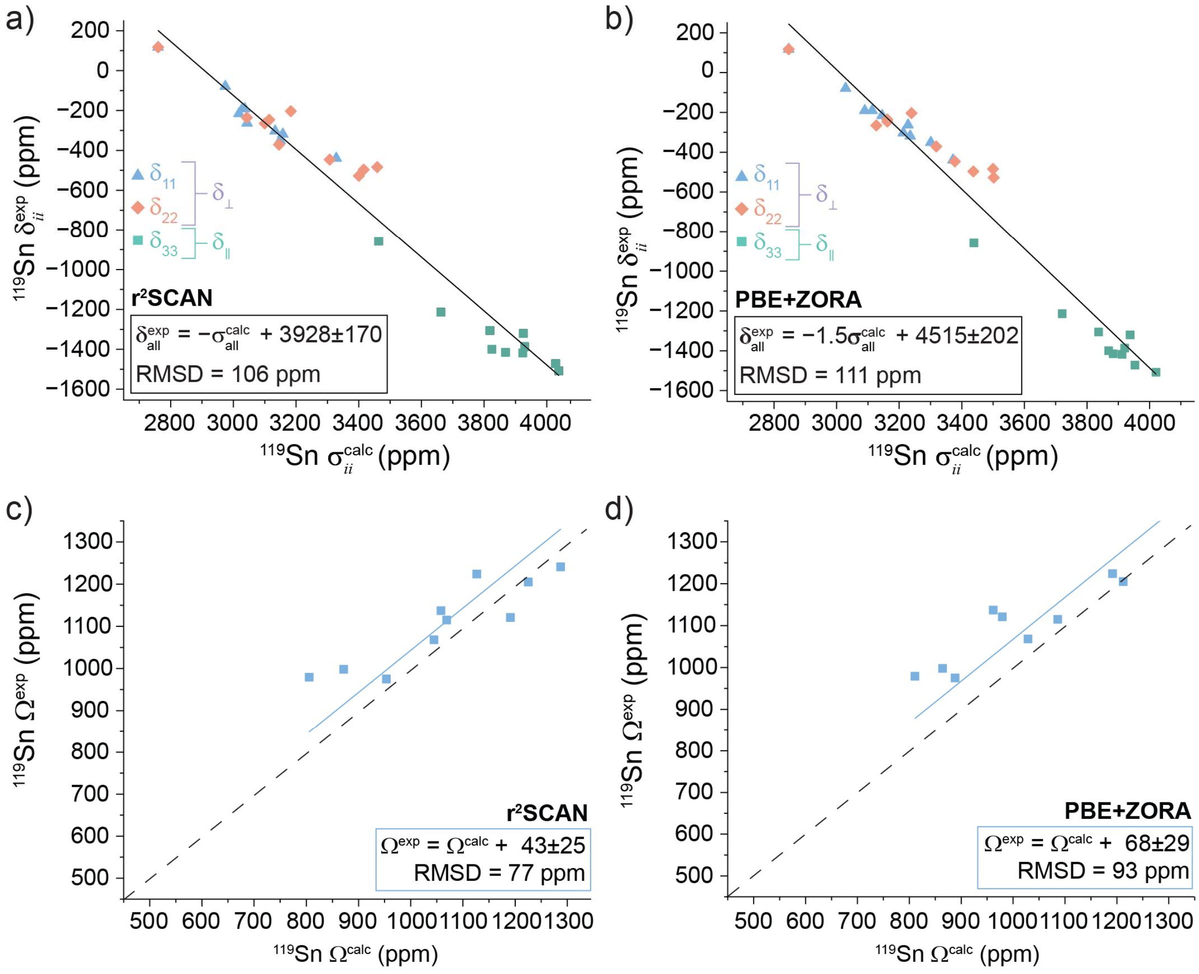

Figure S10: Plot of experimental ^{119}Sn chemical shift principal components against calculated magnetic shielding components obtained from (a) non-relativistic (r^{2}SCAN) and (b) scalar-relativistic (PBE+ZORA) DFT calculations. Linear regressions fitted with variable slopes are shown in black, yielding scaling factors of (a) 1.35 and (b) 1.50. (c,d) Experimental ^{119}Sn spans plotted against calculated ^{119}Sn spans derived using the linear fits of the principal components shown in panels (a,b). (c,d) Linear fits of experimental ^{119}Sn span against calculated span with a fixed slope of 1 are shown in blue.

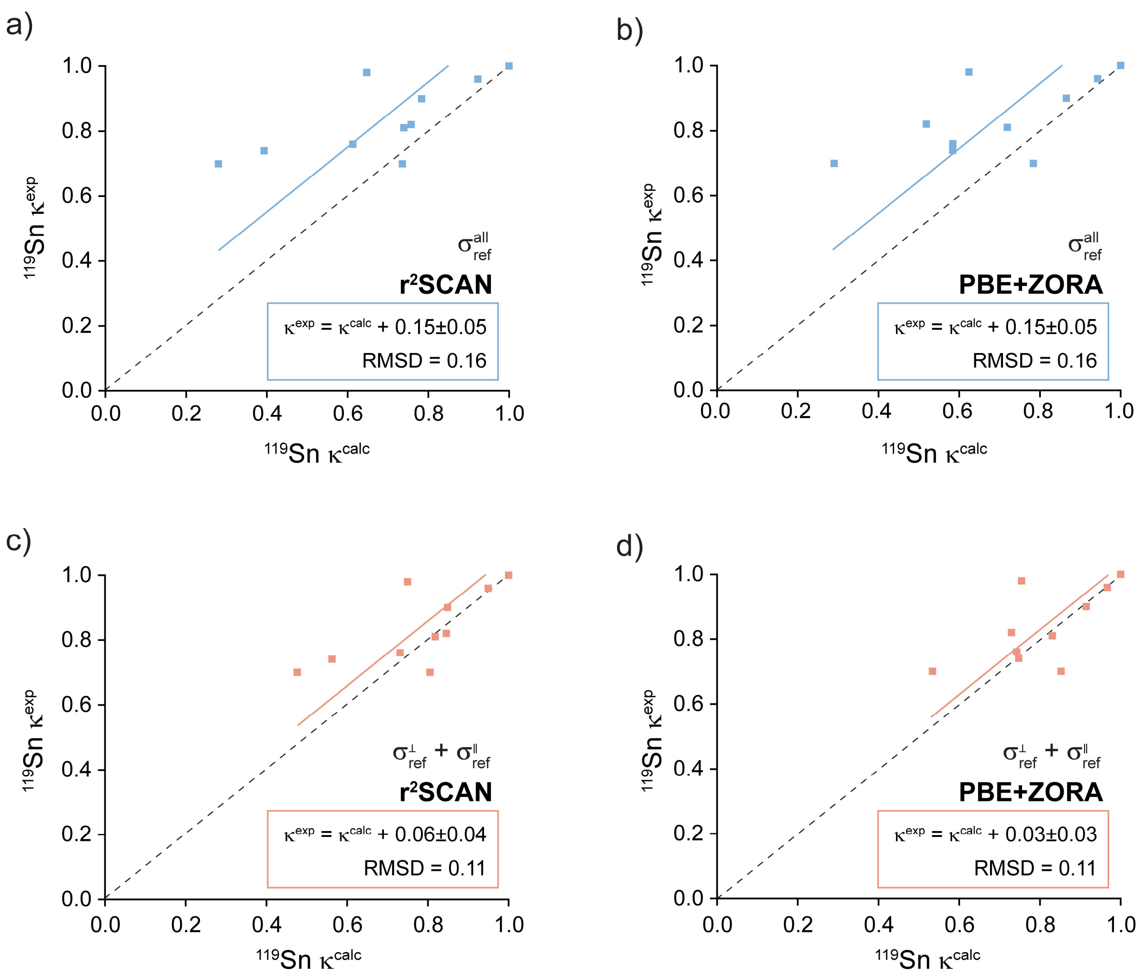

Figure S11: Correlation between calculated and experimental ^{119}Sn skews (κ^{calc} *vs* κ^{exp}) from non-relativistic (r^{2}SCAN) and scalar-relativistic (PBE+ZORA) DFT calculations. (a,b) κ^{calc} values referenced using a single reference shieldings (σ_{ref}) derived from the linear regression of calculated isotropic shieldings against experimental isotropic shifts (σ_{iso}^{calc} *vs* δ_{iso}^{exp}; Table 3). (c,d) Plots of κ^{calc} *vs* κ^{exp} with calculated skew values derived using two reference shieldings for principal components parallel and perpendicular to the ^{119}Sn lone pair, i.e. $\sigma_{\parallel}^{calc}$ *vs* $\delta_{\parallel}^{exp}$ and $\sigma_{\perp}^{calc}$ *vs* $\delta_{\perp}^{exp}$ (Table 3). Dashed black line shows $\kappa^{calc} = \kappa^{exp}$ and blue lines show linear regressions (fixed slope = 1).

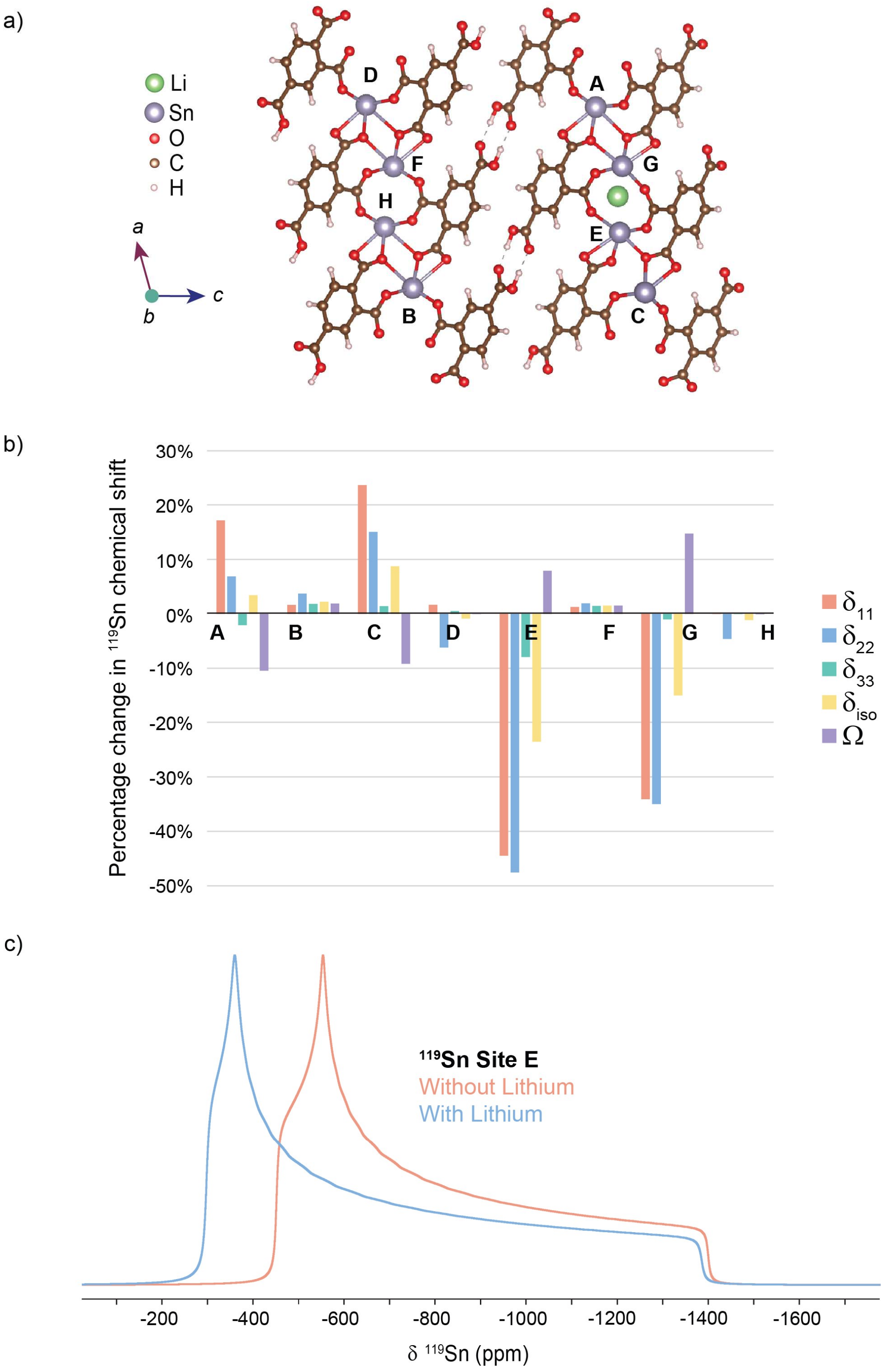

Figure S12: a) Geometry optimized structure of $Li_{0.125}Sn$(H-1,2,4-BTC) in the *ac* plane. DFT calculations were performed using PBE+ZORA, with NMR calculations being performed on a relaxed lithiated structure attained from geometry optimization of the relaxed Sn(H-1,2,4-BTC) structure with a lithium ion inserted between sites E and G. b) Percentage change in calculated ^{119}Sn chemical shifts at the various Sn sites between lithiated and non-lithiated relaxed structures. The distinct ∥ and ⊥ regressions were used to convert shielding to shift, see main text. c) Simulated ^{119}Sn NMR spectra of site E demonstrating how the ^{119}Sn chemical shift anisotropy differs after the addition of a Li ion to the structure.

The greatest change in ^{119}Sn parameters is seen for sites E and G, which have their lone pairs pointing towards the Li^+ cation. Smaller changes are seen for sites A and C within the same chain, while sites B, D, F, and H in the other (non-lithiated) chain show negligible difference (<5 %). This demonstrates that ^{119}Sn NMR is sensitive to the local effect of Li intercalation. The neighbouring sites E and G show a ~10% increase in the ^{119}Sn CSA, clearly observable in the simulated spectrum, which is due to interaction of the Sn(II) lone pairs with the Li^+ cation.
